# Nonlinear Molecular Electronic Spectroscopy via MCTDH Quantum Dynamics: From Exact to Approximate Expressions

**DOI:** 10.1021/acs.jctc.2c01059

**Published:** 2023-03-24

**Authors:** Francesco Segatta, Daniel Aranda Ruiz, Flavia Aleotti, Martha Yaghoubi, Shaul Mukamel, Marco Garavelli, Fabrizio Santoro, Artur Nenov

**Affiliations:** † Dipartimento di Chimica Industriale “Toso Montanari”, University of Bologna, Viale del Risorgimento, 4, 40136 Bologna, Italy; ‡ ICMol, Universidad de Valencia, Catedrático José Beltrán Martínez, 2, 46980 Paterna, Spain; § Istituto di Chimica dei Composti Organometallici (ICCOM-CNR), Area della Ricerca del CNR, Via Moruzzi 1, I-56124 Pisa, Italy; ∥ Department of Chemistry and Department of Physics and Astronomy, University of California, Irvine, California 92697, United States

## Abstract

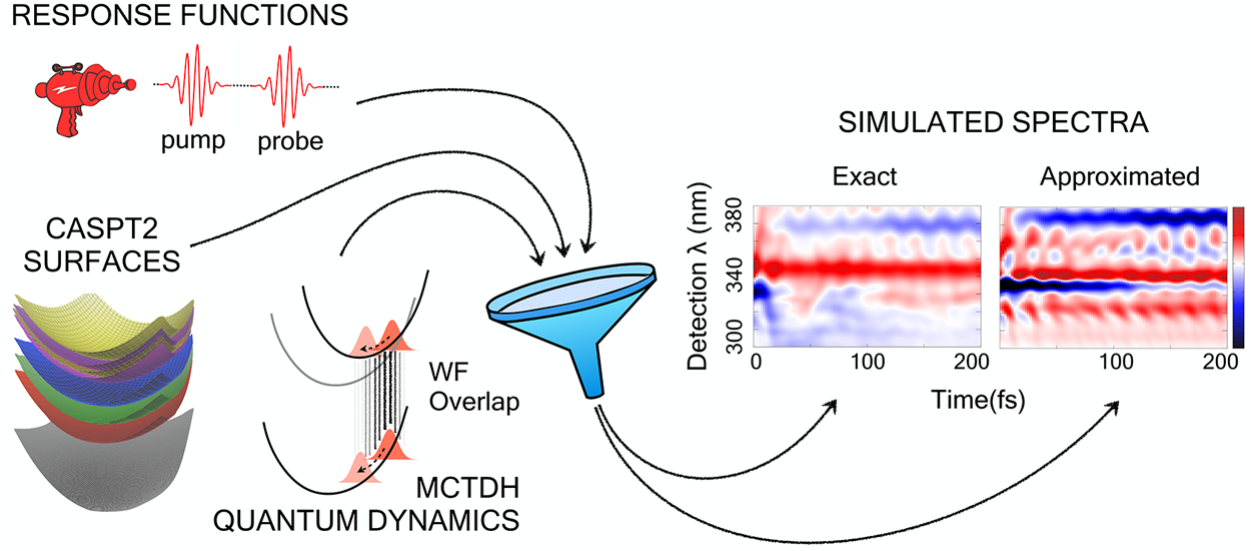

We present an accurate and efficient approach to computing the linear and nonlinear optical spectroscopy of a closed quantum system subject to impulsive interactions with an incident electromagnetic field. It incorporates the effect of ultrafast nonadiabatic dynamics by means of explicit numerical propagation of the nuclear wave packet. The fundamental expressions for the evaluation of first- and higher-order response functions are recast in a general form that can be used with any quantum dynamics code capable of computing the overlap of nuclear wave packets evolving in different states. Here we present the evaluation of these expressions with the multiconfiguration time-dependent Hartree (MCTDH) method. Application is made to pyrene, excited to its lowest bright excited state *S*_2_ which exhibits a sub-100-fs nonadiabatic decay to a dark state *S*_1_. The system is described by a linear vibronic coupling Hamiltonian, parametrized with multiconfiguration electronic structure methods. We show that the ultrafast nonadiabatic dynamics can have a remarkable effect on the spectral line shapes that goes beyond simple lifetime broadening. Furthermore, a widely employed approximate expression based on the time scale separation of dephasing and population relaxation is recast in the same theoretical framework. Application to pyrene shows the range of validity of such approximations.

## 1 Introduction

Time-resolved nonlinear optical spectroscopy provides a wealth of information regarding the photophysical and photochemical steps employed by molecular systems to either dissipate (as in the case of DNA) or exploit (as in the case of light-harvesting systems) the energy absorbed from light. The interpretation of the time-resolved spectra is by no means a simple task as the detected signal arises from the interplay of the electronic structure of the chromophore, the coupling of the electronic and nuclear degrees of freedom, the influence of the environment, and the properties of the light pulses utilized to initiate and probe the photoinduced dynamics. First-principles spectroscopy simulations from atomistic dynamics governed by the laws of quantum mechanics and electrodynamics can provide invaluable insight and help disentangle the contribution of the various microscopic players that eventually build the measured spectrum. Consequently, a great deal of effort has been invested in developing theoretical spectroscopy techniques.

From a theoretical point of view, the third-order polarization *P*^(3)^(*t*) is the quantity of interest for such simulations. Two conceptually different approaches for computing *P*^(3)^(*t*) exist: nonperturbative and perturbative.^1−5^ In the nonperturbative approach, the interaction with the electric field is explicitly included in the (time-dependent) Hamiltonian that drives the dynamics of the system. No assumptions regarding the pulses’ shape, duration, and intensity are made, allowing, for example, to consider the effect of strong fields and overlapping pulses while requiring a typically expensive explicit propagation of the coupled field–matter Hamiltonian, which should be repeated multiple times to extract the desired signal from the total polarization and also for different pulse configurations.^5,6^

The alternative approach, developed by Mukamel et al., is based on a perturbative expansion of the density matrix in terms of the light–matter interaction.^7,8^ The polarization *P*^(3)^(*t*) is recast, separating the properties of the electromagnetic field from the system response to impulsive interactions with the field. These induce a sudden change in the system electronic state, followed by a field-free propagation driven by the sole (time-independent) molecular Hamiltonian. Applications of such a response function formalism for nonlinear spectroscopy simulations of molecular systems are numerous, ranging from isolated molecules in the gas phase to multichromophore aggregates in complex environments.^9−16^ In passing we note that an intermediate approach, proposed by Domcke and co-workers, also exists: the equation-of-motion phase-matching approach (EOM-PMA),^6^ which has provided an efficient method to obtain the third-order polarization at a given phase-matching direction in the case of weak fields (explicitly included in the propagation) through a perturbative procedure.

The (response function) perturbative approach is convenient not only because the field-induced dynamics can be computed only once, taking into account many possible pulse shapes as a postprocessing step, but also because exact analytical solutions for the response function exist when the electronic states are uncoupled and vibrations are described by independent displaced harmonic oscillators. Recently, analytical solutions have also been derived for the more general cases of mode mixing^17^ and anharmonicities.^18^ While the harmonic approximation has been proven to be reliable in many molecular systems, neglecting the coupling among the various electronic states does not allow to describe interstate “transport” processes (i.e., the nonadiabatic processes that induce a change in the system electronic state, such as internal conversion through conical intersections, intersystem crossings, and energy transfer in multichromophoric systems). The third-order response can be recast to take into account transport in an approximate way by treating differently the field-free evolution of populations and coherences (i.e., the diagonal and off-diagonal elements of the density matrix). In particular, when the system resides in a population, transport is accounted for in the form of time-dependent weighting factors that tune the intensity of the state-specific spectral contributions. Instead, when the system is in coherence, transport is assumed to merely contribute to the homogeneous broadening of said contributions. This approach is formally motivated when a separation of time scales holds (i.e., when populations exhibit much longer lifetimes (hundreds of femtoseconds and beyond) compared to the duration of coherences which are quenched on the time scale of a few tens of femtoseconds due to dephasing). Despite the aforementioned separation of time scales not being rigorously justified for ultrafast transport processes, analytical third-order response expressions, parametrized with high-quality quantum mechanics (QM) data, have allowed the successful simulation of nonlinear spectroscopies for a number of organic molecules such as azobenzene,^19^ polyaromatic hydrocarbons,^20,21^ and thio-nucleobases,^22^ in remarkable agreement with experiment.

In the present contribution, we would like to move beyond such an approximate treatment of the response function and underlying nonadiabatic dynamics. On the one hand, this is necessary to describe ultrafast nonadiabatic processes where the above-mentioned separation of time scales is not justifiable. On the other hand, this would also provide a useful benchmark for approximations, allowing an assessment of their limitations and regime of validity.

We considered an explicit numerical quantum dynamics approach, namely, the multiconfiguration time-dependent Hartree (MCTDH).^23−25^ MCTDH allows to run nonadiabatic dynamics, and it is extremely efficient on potential energy surfaces that have some simple (low-order polynomial) functional form, allowing the avoidance of drastic truncation of the dimensionality.^26,27^ The MCTDH scheme has been previously utilized in the nonperturbative approach to computing transient absorption (TA) and two-dimensional (2D) electronic spectroscopy.^4,28,29^ Recently, applications of the Gaussian-based MCTDH (G-MCTDH) approach to describe nonlinear spectroscopy have also been demonstrated.^30^ The ingredients required to parametrize the molecular Hamiltonian are similar to those utilized in the approximate expressions described above (knowledge of the electronic and nuclear degrees of freedom and their coupling), with the addition of the interstate couplings, which may also be obtained from QM calculations. In this work, we present a general expression for the TA signal in the framework of the response function formalism and its specific implementation in Quantics, an MCTDH-based package.^31,32^ We demonstrate the implementation in the example of the *S*_2_ → *S*_1_ internal conversion of the polycyclic aromatic hydrocarbon pyrene, for which we recently presented a linear vibronic coupling (LVC) Hamiltonian parametrized with accurate wave function-based multiconfigurational electronic structure theory.^33^ Other approaches for nonlinear spectroscopy simulation that go beyond the populations/coherences separation exist, such as, to name a few, grid-based quantum dynamics methods (that can typically consider up to 2/3 nuclear degrees of freedom),^34,35^ the hierarchical equations of motion HEOM^36^ (which provide exact results at the expense of handing spectral densities of simplified form), semiclassical path-integral approaches^14^ (which nonetheless rely on different flavors of the semiclassical approximation), and a number of platforms have been developed to perform such simulations.^37−40^

In what follows, we first present the fundamental equations for the first- and third-order response functions in the impulsive limit. We will refer to the highest level of theory as the exact method, meaning that, within a closed quantum system description and the Condon approximation, the response function is obtained by solving the time-dependent Schrödinger equation in a numerically exact way. Then, a series of approximations are presented, leading to the (approximate but analytical) line-shape function expressions. The final equations are labeled as QD (the highest level of theory that can be obtained via a quantum dynamics simulation), QD* (for which the populations/coherences time-scale separation is invoked), and SPEC (the most approximate level that also employs the second-order cumulant expansion of Gaussian fluctuations (CGF) to evaluate the wave packet overlap analytically). We also discuss how we realize these spectroscopy simulations extracting the necessary data from MCTDH nuclear wave packet propagation (similarly labeling the equations as MCTDH, MCTDH*, and SPEC). Subsequently, both the exact and approximate equations for the nonlinear response are adapted to the problem of the *S*_2_ → *S*_1_ internal conversion in pyrene. The linear absorption and TA spectra obtained within the two representations are compared, thereby elucidating the consequences of the approximations adopted.

## 2 Theory

### 2.1 Transient Absorption Signal

We consider the simplest form of third-order nonlinear spectroscopy, TA, in which a system is impulsively excited by a first laser pulse, the pump, and the subsequent dynamics is monitored by a second laser pulse, the probe. The theoretical foundations of nonlinear spectroscopy can be found in refs (7) and (8).

**Figure 1:**
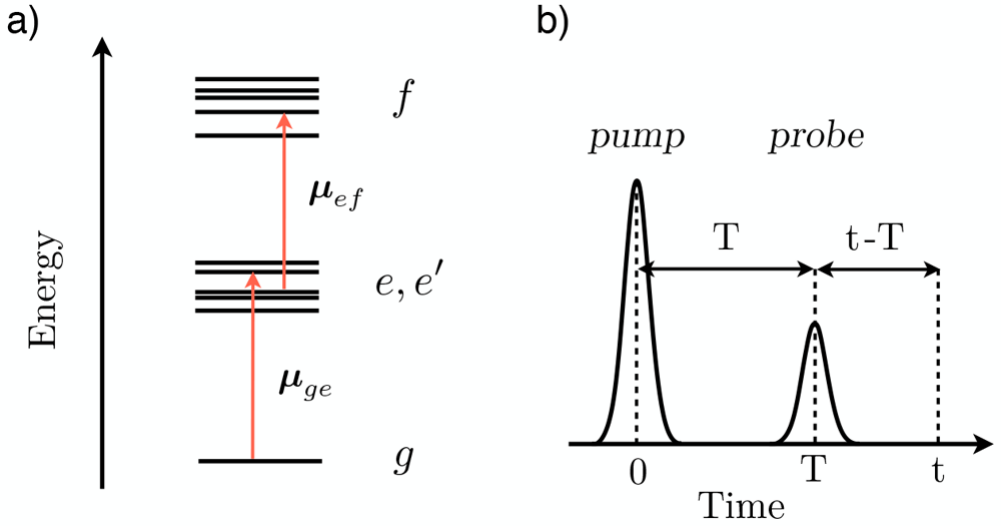
(a) Energy-level scheme and allowed transitions among the GS (*g*), the *e* manifold, and the *f* manifold. (b) Pulse sequence and time delays.

The system, initially in its electronic ground state (GS) labeled *g*, is promoted at time zero to higher-lying electronic states resonant with the pump. These states, together with the states vibronically coupled to them, form the *e* manifold in which the nonadiabatic electronic dynamics occurs (thus called active states). We note that in the most general convention, the *e* manifold comprises all singlet and triplet states participating in internal conversion (IC) and intersystem crossing (ISC) phenomena. The probe pulse, delayed in time by the waiting time *T*, promotes the system to a lower-lying state or to a higher-lying state, giving rise to stimulated emission (SE) or excited-state absorption (ESA), respectively. The GS is assumed to be energetically well separated from the *e* manifold. The manifold of higher-lying states, labeled with *f*, comprises states not involved in the pump-induced nonadiabatic dynamics (thus called spectator states) that are used as spectral fingerprints of the active states (see Figure 1). Overall, it should be clear that the separation of the electronic states in *g*, *e*, and *f* manifolds is determined by the characteristics of the pump and probe pulses.

A sequence of light–matter interactions produces a time-dependent polarization of the electronic density, which is the quantity of interest in spectroscopy, being the source of the detected signal field. In the weak field regime, the light–matter interaction can be treated perturbatively: the polarization can be written as an order-by-order expansion in the light–matter interaction. In the dipole approximation (i.e., when the pulses’ wavelengths are much longer than the dimensions of the system), which is certainly valid in the UV/vis regime, the perturbation reads , with **μ̂** and ***E***(*t*) being the transition dipole moment operator and the electromagnetic field, respectively. The expression for the third-order polarization reads
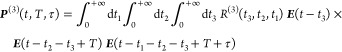
1where the superscript (3) indicates that three light–matter interactions are considered and *R*^(3)^(*t*_3_, *t*_2_, *t*_1_) is the so-called (third-order) response function that encodes the system response to the light-induced perturbations. The third-order polarization depends on the waiting times τ and *T*, denoting the time between the first and second field–matter interactions and between the second and third field–matter interactions. In TA spectroscopy, the system interacts with just two pulses: the (typically strong and short) pump and the (weaker) probe. The first two field–matter interactions both occur beneath the pump pulse envelope so that τ = 0, and separate measurements are performed by changing the delay *T* between the pump and the probe.

The field ***E***(*t*) is given by the sum of the pump and probe pulses, i.e., ***E***(*t*) = ***E***_1_(*t*) + ***E***_2_(*t*) (see Figure 1). Following the assumption we made that the pump pulse arrives first (thus labeled by the subscript 1) and is centered at time *t* = 0 and the probe pulse arrives later and is centered at time *T*, one can rewrite the electric field as 
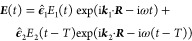
, where *E*_1/2_(*t*) represents the time envelopes and  represents the pulse polarizations. An evaluation of the triple integral of eq 1 is rather costly in the perturbative formulation as in general it requires *N*_1_ × *N*_2_ × *N*_3_ simulations (*N*_2_ × *N*_3_ in the case of TA) of the response (with *N*_*i*_ being the number of grid points in the time domain during the three time intervals). In that regard, it is worth noting the recent effort by Krich and co-workers, who reported a fast evaluation of the triple integral utilizing the convolution theorem and the fast Fourier transform.^41^ In the present work, we adopt the impulsive limit, thus assuming that the pulses are well separated in time and have a duration much shorter than any relevant time scale in the dynamics studied. In the impulsive limit, one can approximate the pulses as δ-like functions so that the third-order polarization becomes equivalent to the response
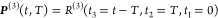
2where *t*_1_ = 0 and *t*_2_ = *T* are the times of arrival of the pump and probe pulses, respectively, and *t*_3_ = *t* – *T* is the time delay between the last field–matter interaction and the detection of the field.

The detected signal can be computed as the Fourier transform of the polarization (i.e., of the response function, with respect to the time delay *t*_3_ = *t* – *T*):
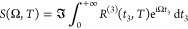
3Practically, this Fourier transform step requires the *t*_3_ interval to be sampled frequently enough to follow the fastest time scales of the system dynamics. Note that large frequency oscillations dictated by the energy gaps between the various electronic states can be conveniently set to zero before the Fourier transform step and reintroduced after it. This is described below.

At this point, it is evident that *R*^(3)^(*t*_3_, *T*) is the main ingredient required to simulate the TA. Therefore, in what follows, we will focus on its evaluation.

### 2.2 Response Functions: The Impulsive Limit

The first- and third-order response functions, written in their most general form,^7^ read
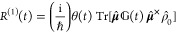
4
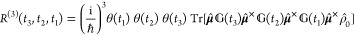
5They contain a sequence of field–matter interactions, captured by the superoperator  that acts on its right as , where • represents a generic operator, and promotes a sudden change in the electronic state in which the system resides, and field-free propagations, captured by the time-evolution superoperator , that describes the system evolution during the various time intervals.  is the initial density matrix (prior to any field interaction), and θ(*t*) is the Heaviside step function.

Let us rewrite eq 5 by unpacking the action of the superoperators  and . The *t*_1_ time interval is set to zero (i.e., ), which implies an ultrashort pump pulse whose time width is shorter than any relevant time scale of the system’s dynamics. One obtains
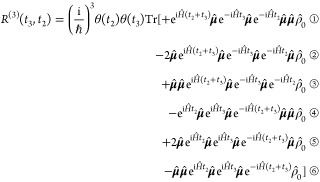
6where we have also made use of the cyclic property of the trace to leave the density matrix on the right of each term. It is apparent that some of the terms are complex conjugates of others; in particular, we note that ① = ⑥*, ② = ⑤*, and ③ = ④*.

Similar (and simpler) operations can also be performed for eq 4, obtaining
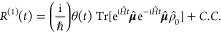
7In what follows, we do not explicitly consider the *C*.*C*. terms of eqs 6 and 7, as these are redundant.^10^ For TA, we will consider the contributions arising from the phase-matched direction corresponding to the TA signal.^7^

A density matrix formulation of the response function is typically employed as it allows one to describe open-quantum systems (i.e., systems coupled to an environment). Such a coupling is typically treated through its spectral density parametrized phenomenologically^4^ or from dynamics simulation under the influence of the solvent^42,43^ which models the continuum of low-frequency (highly anharmonic) bath modes. Here, we treat the dynamics of the molecular system independently from the environment (i.e., as a closed quantum system), which is perfectly suited for gas-phase spectroscopy simulations. In the current derivation, we chose to mimic the environment effect by applying a phenomenological dephasing term that damps the response function and introduces a homogeneous contribution to the total broadening, adopting a wave function formulation.

### 2.3 Response Functions: Wave Function Formulation

Let us now describe how the response function can be rewritten in terms of the wave-packets obtained in a QD simulation. At a given time *t*, the molecular wave function can be written as
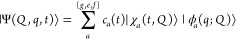
8where |χ_*a*_(*t*, *Q*)⟩ represents the normalized nuclear wave packet evolving on the *a*th electronic state  which can be represented in a time-dependent or time-independent basis.^23^*c*_*a*_(*t*) is the amplitude of the *a*th electronic state defined here as . ρ_*a*_(*t*) is the population of the *a*th electronic state so that  represents a real-valued time-dependent factor which scales the normalized nuclear wave packet χ_*a*_(*t*, *Q*) according to the electronic state population. (An alternative notation assimilates *c*_*a*_(*t*) in the definition of the wave packet.^23^) *E*_*a*_^0^ is the *a*th electronic state energy at a given reference (e.g., at the Franck–Condon point). *q* and *Q* denote the electronic and nuclear degrees of freedom, respectively. The index *a* runs over the three manifolds *g*, *e*, and *f* shown in Figure 1. The condition ⟨Ψ(*Q*,*q*,*t*)|Ψ(*Q*,*q*,*t*)⟩ = 1 holds for each time *t*. The electronic wave function  is conveniently rewritten as |*g*⟩, |*e*⟩, and |*f*⟩ for the GS and for states of the *e* and *f* manifolds, respectively.

The molecular Hamiltonian reads
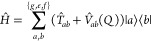
9where  and  denote the kinetic and potential energy terms, respectively. Explicit expressions for  and  in the diabatic representation are given in section S1 of the SI. It is common to work in either the adiabatic (potential term diagonal) or diabatic (kinetic term diagonal) basis. Below we adopt the diabatic formulation, which has certain advantages with respect to the parametrization. In particular, in the diabatic picture the electronic wave functions become (ideally) independent of the nuclear coordinates and the corresponding potential energy surfaces (i.e., the potential energy term) can be approximated through a low-order Taylor expansion relying on quantum-mechanical data from a handful of reference geometries (see section 3). This idea is at the heart of the LVC model which utilizes a quadratic expansion of the diagonal terms of the potential energy operator *V*_*ab*_ which encode the electronic state energies and the coupling to the nuclear degrees of freedom, termed intrastate coupling. The off-diagonal terms which describe the coupling (mixing) between electronic states, thus termed interstate coupling, are assumed to be linear functions of the nuclear coordinates. It is further assumed that all potential energy surfaces share the same set of normal modes and frequencies, an approximation known as the displaced harmonic oscillator (DHO). The LVC model is the simplest Hamiltonian capable of describing conical intersections and is widely used to model the ultrafast nonadiabatic dynamics of multidimensional molecular systems.^26,27^ In passing, we note that the equations presented for the spectroscopy are valid for any form of the molecular Hamiltonian.

The transition dipole moment **μ̂** is given by
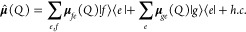
10and describes the light–matter interaction that couples states from the *g*, *e*, and *f* manifolds. Here it is assumed to be coordinate-independent (the Condon approximation, i.e., **μ**_*ij*_(*Q*) = **μ**_*ij*_). As previously noted, in a diabatic representation, this is a quite reasonable approximation since diabatic states are defined to be virtually independent of the nuclear coordinates.

**Figure 2:**
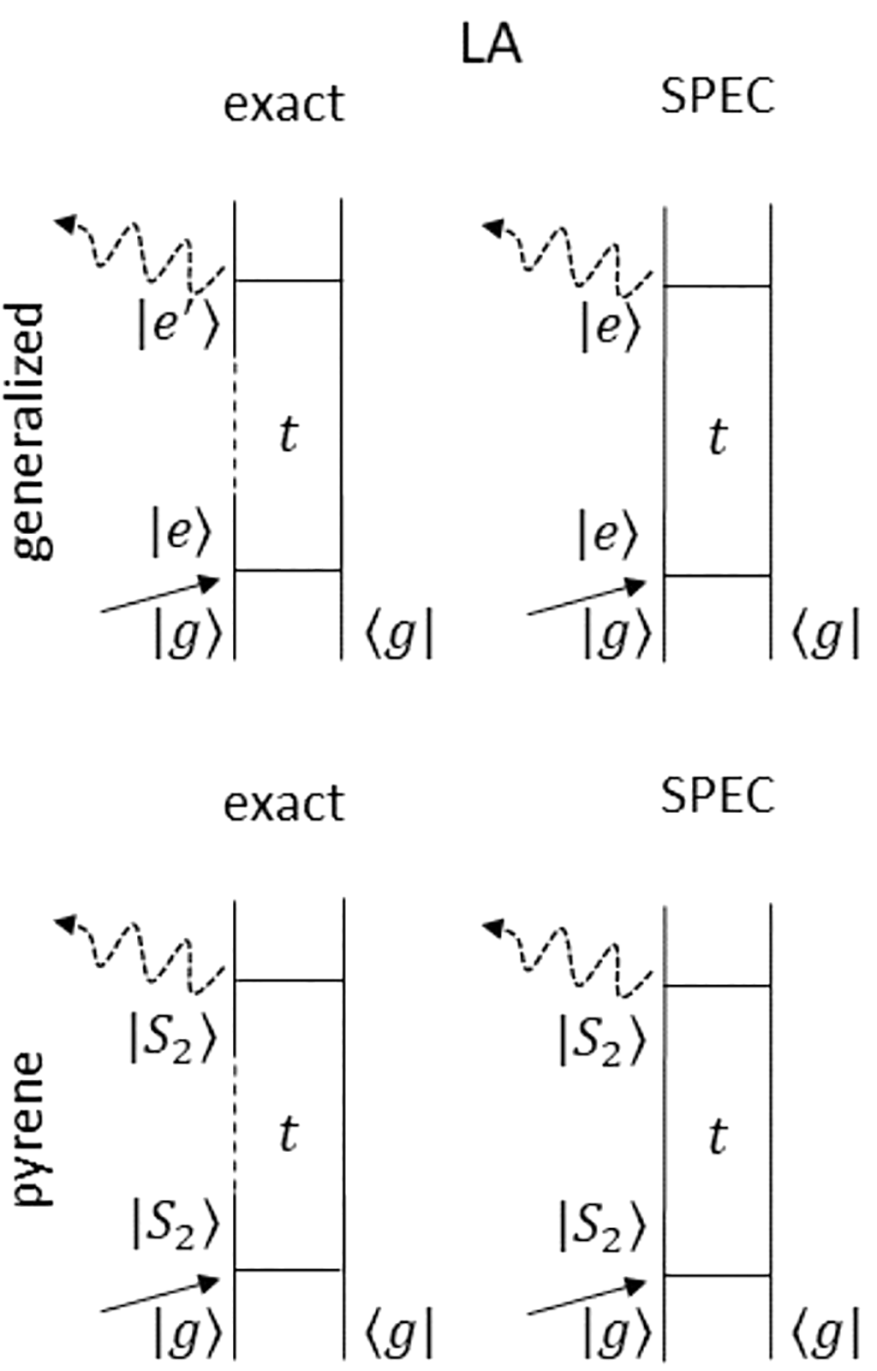
(Top) Feynman diagrams for the first-order linear absorption signal generated with the exact formulation (eq 12) and SPEC approximation (eq 17). (Bottom) Feynman diagrams for the exact and SPEC approximations applied to the special case of pyrene (eq 20). Dashed lines represent intervals in which the  nonadiabatic dynamics is turned on. In the special case of pyrene, |*e*⟩ refers to *S*_2_, whereas  can refer to either *S*_2_ (fraction of the population remaining in the initial state after the interval *t*) or *S*_1_ (fraction of the population transferred). Due to the vanishing  electric-dipole coupling, only the fraction in *S*_2_ contributes to the signal.

For a closed quantum system, eq 7 can be recast in terms of the wave function without loosing generality, obtaining
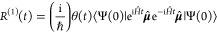
11where |Ψ(0)⟩ is the (initial) GS wave function, and we dropped the explicit notation of the *Q* and *q* dependence in the wave function. After inserting eqs 8 and 10 and considering the orthonormality of the electronic states, one obtains
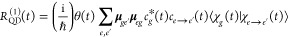
12where the subscript QD (quantum dynamics) refers to the fact that this expression can be evaluated via a quantum dynamics simulation. In the above expression, the term in the brackets describes the time-dependent overlap between the nuclear wave packets evolving on different electronic potential energy surfaces, which are dynamically weighted by the electronic amplitudes. In passing, we note that since the wave packet is in equilibrium in the GS, its sole dynamics rely on a time-varying phase factor  and , where ϵ_0_ is the sum of the zero point vibrational energies along all modes. Thereby, the notation *c*_*a*→*b*_(*t*) (and χ_*a*→*b*_(*t*)) refers to the nuclear wave packet evolving from the electronic state *a*, where it was initially created at time zero, to the electronic state *b* at time *t*, driven by the nonadiabatic dynamics (*a* and *b* belong to the *e* manifold). Note that the double summation runs over both *a* ≠ *b* and *a* = *b* cases, with the second case indicating the part of the wavepacket that at time *t* is still found in the same electronic state in which it was created. The Feynman diagram representation of such a response function is reported in Figure 2 (top).

**Figure 3:**
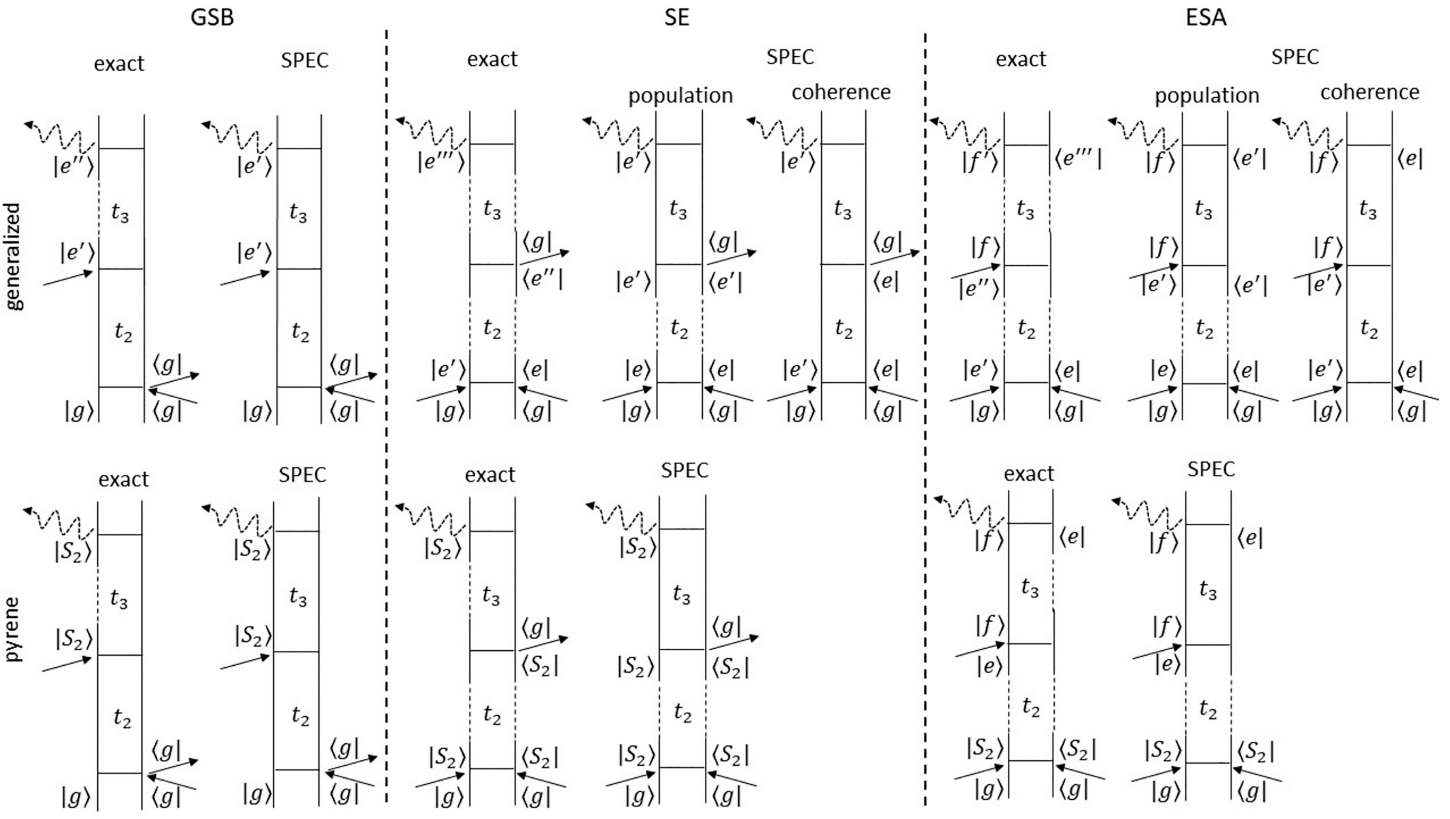
(Top) Feynman diagrams for third-order nonlinear TA signals generated with the exact formulation (eq 13) and SPEC approximation (eq 19). (Bottom) Feynman diagrams for the exact and SPEC approximations applied to the special case of pyrene (eq 21). Dashed lines represent intervals in which the nonadiabatic dynamics is turned on. In the special case of pyrene,  can refer to either *S*_2_ (fraction of the population remaining in the initial state after the interval *t*) or *S*_1_ (fraction of the population transferred). Vanishing diagrams (due to null dipole coupling between specific pairs of states) are not reported.

Following similar steps, the nonlinear response function of eq 6 (dropping the prefactor 2(i/*ℏ*)^3^θ(*t*_2_)θ(*t*_3_)) eventually reads
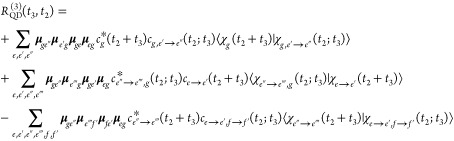
13Here, the first term of the equation is referred to as ground-state bleaching (GSB), the second, as stimulated emission (SE), and the third (which involves states of the *f* manifold), as excited-state absorption (ESA). The Feynman diagram representation of such a response function is reported in Figure 3 (top). As explained above, the various subscripts keep track of the nonadiabatic history of the wave packet evolution. As an example, in the first term of eq 13,  encompasses the following information about the wave packet: the nuclear wave packet evolved on the *g* state for a time interval *t*_2_ and then was suddenly promoted (by an interaction with the field) to the *e* state, and the dynamics during the *t*_3_ time interval leads it to the *e*″ state. Since the *c*_*g*_(*t*) time-dependence is trivial (just a phase factor), the first term of the equation can be further simplified as .

As previously noted, we can still include (to some extent) the effect of the coupling between the molecular system and the environment via a dephasing term, which can either be a Gaussian or an exponential term. We choose the former functional form, and the first-order response therefore becomes
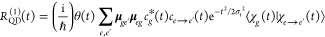
14where σ_*t*_ dictates the dephasing time scale (that is assumed here to be the same for all states from the *e* manifold). Similarly, one can include such a phenomenological dephasing term in the nonlinear response function (eq 13).

### 2.4 Response Functions: Approximate Expressions

Equations 12 and 13 are completely general formulations of the first- and third-order response functions for a closed quantum system subject to impulsive interaction with nonoverlapping δ-pulses within the Condon approximation. Their solution requires explicit propagation of the nuclear wave packet (e.g., by means of the MCTDH approach). In this section, we adopt several approximations giving rise to analytical expressions for the linear and nonlinear response functions that have found widespread application.^7^

The key approximation is that of explicitly taking into account the electronic dynamics only when the system is in a population (i.e., when the bra and ket wave packets reside in the same electronic state at a given instant in time). The underlying physical motivation for such an approximation leverages the separation of time scales: the dephasing time scale of the coherences is assumed to be much shorter than the time scale of the population dynamics. Consequently, the product of amplitudes *c*_a_^*^(*t*)*c*_*b*_(*t*) (with *a* and *b* being generic labels for electronic states) changes according to the nonadiabatic dynamics only when the bra and ket wave packets are in a population (i.e., *a* = *b*), which occurs only during the *t*_2_ interval. When *a* ≠ *b*, the dephasing term is assumed to quench the signal so rapidly that it is immaterial to explicitly consider nonadiabatic effects. This approximation has to be taken with caution when treating ultrafast nonadiabatic dynamics occurring on the same time scale of the dephasing (i.e., on the order of a few tens of femtoseconds).

Under the hypothesis of time scale separation between dephasing and population transfer, the first-order response reads
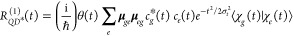
15and the double summation over *e*, *e*^′^ is substituted by the simple *e* summation since *e* can no longer transform into *e*^′^. We further note that *c*_g_^*^(*t*)*c*_*e*_(*t*) is simply equal to an oscillating phase term, namely, , where ℏω_*eg*_^0^ is the adiabatic electronic energy gap between states *e* and *g*. The effect of the nonadiabatic dynamics can be approximately reintroduced at the level of the *c*_*e*_(*t*) coefficient: in the absence of transport, , but one might still account for the finite lifetime of the *e*th state, exploiting the knowledge of its population dynamics (i.e., ). Thus, one obtains
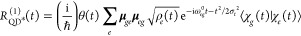
16A further approximation consists of describing the transport among the various electronic states via a set of Pauli master equations, for which , with τ_*e*_ being the *e*th state lifetime. Finally, in the absence of transport, the  term is simply the overlap between two wave packets that oscillate adiabatically on their respective potential energy surfaces and therefore, in the framework of harmonic potential energy surfaces (i.e., the DHO model), does not need any explicit quantum dynamics to be evaluated. Indeed, analytical expressions for such an overlap term can be written within the so-called second-order CGF expansion,^7^ obtaining the final expression labeled with the subscript SPEC and given by
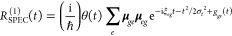
17where we used the compact notation ξ_*ab*_ = ω_*ab*_ – i/2τ_*ab*_ (and we also defined τ_*ab*_^–1^ = τ_*a*_^–1^ + τ_*b*_^–1^, with τ_*g*_^–1^ = 0), ℏω_*ab*_ is the vertical energy gap between states a and b, and *g*_*ge*_(*t*) is the analytical line-shape function from CGF^7^ (see section S5 in the SI). In passing, we note that the truncation to second order of the CGF expansion is not an approximation within the DHO model (for which all cumulants higher than the second vanish identically).^8^ Therefore, within the harmonic approximation, the SPEC equations are expected to provide similar results to the QD* equations when the transport process can be described by the Pauli master equations.

Similar reasoning holds for the nonlinear response function. The time scale separation between dephasing and population transfer described above leads to
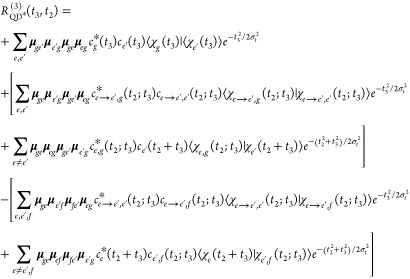
18For the SE and the ESA contributions, we differentiate population (first term in the square brackets) from coherence (second term in the square brackets) contributions, depending on whether the system is subject to population transfer during interval *t*_2_. This formal grouping of pathways shows that the dynamics in the coherences is purely adiabatic. As a consequence, the expressions for the overlaps in the coherences can be resolved analytically by means of second-order cumulant expansion (CGF). The populations instead keep track of the (nonadiabatic) history of the wave packet during the *t*_2_ interval. Thus, their correct implementation would require explicit quantum dynamics to be performed. To avoid that, one further approximation needs to be adopted: we assume that the wave packet is created in the arrival state *e*′ already at *t*_2_ = 0 where it propagates adiabatically during the *t*_2_ interval. In this approximation, the expressions for the overlaps in the population terms adopt the forms  and , which enables their analytical evaluation by means of CGF. After a few steps similar to those employed for the first-order response, one obtains
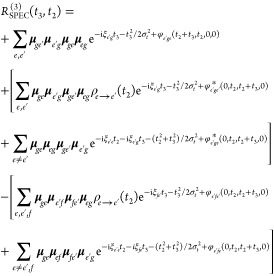
19where φ_*cba*_(τ_4_, τ_3_, τ_2_, τ_1_) represents the multidimensional phase functions resulting from applying CGF to the overlaps (see section S5 in the SI). As done for the first-order response, the effect of the population dynamics in the *e* manifold during *t*_2_ can be accounted for by introducing , which acts as a time-dependent weighting factor modulating the intensity of a state-specific SE and ESA. In particular, for *e* = *e*′ it gives rise to signals due to the fraction of the population that is found in the initially excited state *e* at *t*_2_, whereas for *e* ≠ *e*′ it gives rise to signals from all other *e*′ states populated nonadiabatically. ρ_*e*→*e*′_(*t*_2_) can be obtained by solving the Pauli master equation  with  being the population rate matrix whose diagonal and off-diagonal elements  and  are the inverse lifetime of state *a* and the negative of the transfer rate from state *a* to *b*, respectively.^7^

It should be noted that initiating at *t*_2_ = 0 adiabatic dynamics directly in each of the *e*′ states without explicitly considering the nonadiabatic history of the wave packet introduces biases. These are: memory erasure of the vibrational dynamics in previously visited electronic states and violation of the conservation of energy (a wave packet passing from a higher-energy state to a lower-energy one will, in general, have a larger kinetic energy with respect to a wave packet directly created in the lower state). This has several repercussions on the spectral line shapes: the simulated signals may present incorrect amplitudes, phases, and shapes for signals from states populated via the transport process.

Finally, we also note that the number of overlap terms can be reduced by selecting only pairs of states coupled through a non-negligible dipole moment, **μ**_*ab*_. For example, note that the second ESA term in eqs 18 and 19 is different from zero if and only if the state *f* is dipole-allowed from both *e* and *e*′. We exploit this to simplify the equations in the specific case of the pyrene molecule.

### 2.5 Practical Implementation of the Exact and Approximate Response Functions

#### 2.5.1 MCTDH

Solving eqs 12 and 13 requires a model Hamiltonian in order to perform the wave packet propagation and to collect quantities such as amplitudes and nuclear wave function overlaps.

In the current work, we present an implementation of such equations based on wave packet propagation carried out with the MCTDH method implemented in the Quantics package.^32^ MCTDH expands the nuclear wave packet χ_*a*_(*t*, *Q*) (eq 8) in the basis of time-dependent functions, known as single particle functions (SPFs). The SPFs in turn are expanded on a primitive (time-independent) grid. The use of time-dependent basis functions confers a greater flexibility in the description of the wave packet compared to approaches relying on time-independent basis function expansions, thus allowing work with low-dimensional expansions. The SPFs and expansion coefficients are propagated according to equations of motion that provide a variational solution to the time-dependent Schrödinger equation. The MCTDH formulation is optimal for Hamiltonians of a simple form, such as products of one-coordinate operators. The multidimensional LVC model Hamiltonian falls under this definition, allowing us to consider a few dozen nuclear degrees of freedom in the single-layer implementation of MCTDH and several hundred in the multilayer extension.^44−47^ Despite the attribute “model”, vibronic coupling Hamiltonians have found widespread use in describing real problems. They are particularly well suited for studying IC and ISC dynamics on the time scale of a few hundred femtoseconds in rigid systems. Fields of applications range from polycyclic aromatic fused rings^33,48−50^ and DNA nucleobases^51−54^ to transition-metal complexes^55−58^ to molecular aggregates.^59−62^

**Figure 4:**
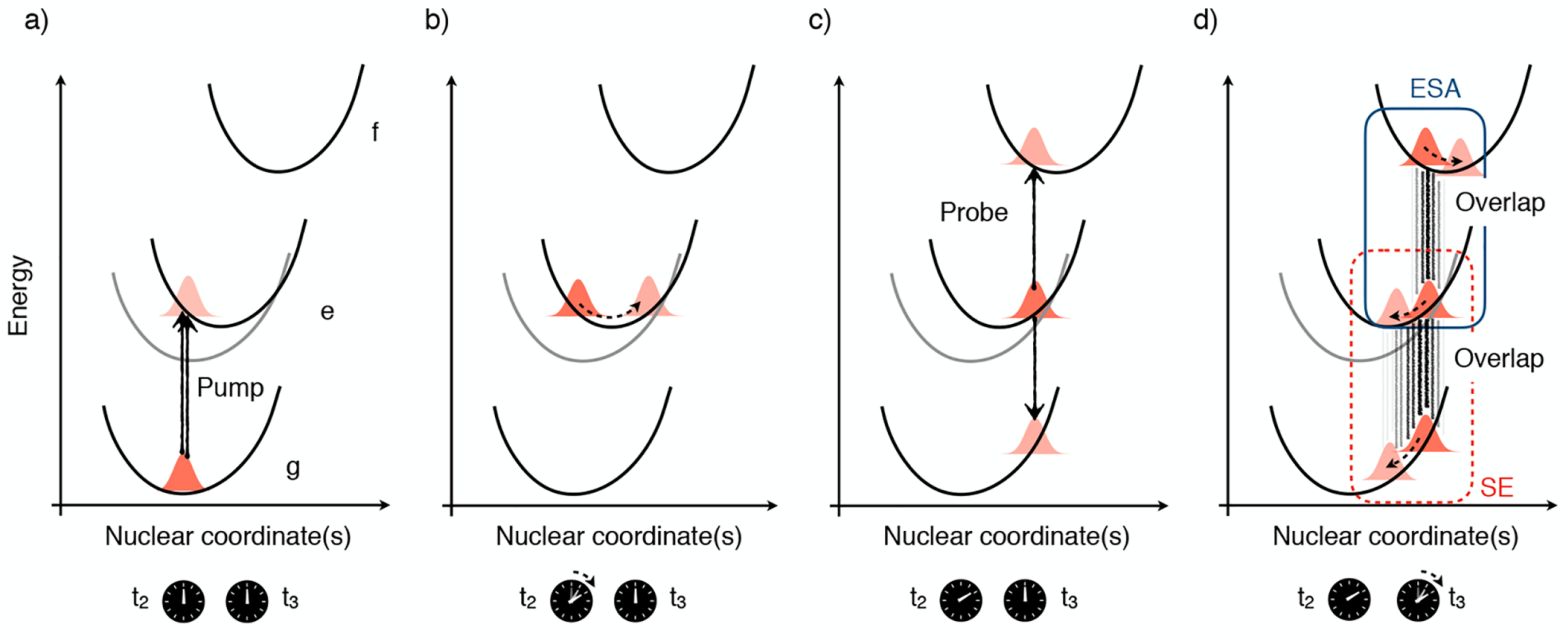
Workflow of the MCTDH-spectroscopy approach: (a) system preparation in the *e* manifold (corresponding to a vertical projection in the impulsive limit); (b) field-free propagation along *t*_2_; (c) projection of the wave packet to the *g* and *f* manifolds; and (d) field-free propagation along *t*_3_ and extraction of the nuclear wave packet overlap in equal (*t*_3_) time intervals.

The ingredients required to compute the nonlinear response with eq 13 are (a) the electronic structure of the *g*, *e*, and *f* manifolds; (b) the coupling of the electronic states to the vibrational degrees of freedom, termed intrastate couplings; (c) the coordinate-dependent interstate couplings; and (d) the coordinate-independent (in the Condon approximation) TDM **μ**_*ab*_. The quantities can be obtained through QM calculations with one or more reference geometries. The energies and couplings are used to parametrize the model LVC Hamiltonian (eq 9) utilized by Quantics to propagate the system.^33^ The time-dependent electronic amplitudes *c*_*a*_(*t*) and nuclear overlaps  obtained from the dynamics, together with the TDMs, are then used to compute the transient signals following eq 13. In practice, the following workflow is adopted (Figure 4):

- The dynamics is initiated by defining the initial amplitudes of the molecular wave function (eq 8, Figure 4a). Thereby, several states can be simultaneously populated provided they fall under the spectral envelope of the pump and exhibit finite dipole coupling to the GS. Note that, as depicted in Figure 3, both populations and coherences can be created if the pulse bandwidth is resonant with multiple states.
- The dynamics driven by the Hamiltonian (eq 9) is propagated in the *e* manifold for the time interval *t*_2_ (Figure 4b).
- In equal intervals along the *t*_2_ axis and for each state of the *e* manifold, copies of the wave packet χ_*e*_(*t*_2_) are projected onto the states of the *g* and *f* manifolds exhibiting non-negligible dipole moments by assigning them the same amplitudes and SPFs (Figure 4c). In this sense the wave packet created in each state of the *g* and *f* manifolds is a replica of χ_*e*_(*t*_2_). This procedure is implemented much more straightforwardly by adopting a multiset formalism for the MCTDH wave function, where each electronic state is assigned an independent number of single-particle functions. Notice that such a projection needs to be repeated frequently enough (every few femtoseconds) to capture the relevant (*t*_2_-dependent) oscillatory features of the vibrational dynamics.
- The total wave packet is renormalized and the replicas are propagated for a time interval *t*_3_, generally a few tens/hundreds of femtoseconds (Figure 4d).
- In a postprocessing step, the overlaps  are obtained in equal intervals along the *t*_3_ axis by computing the expectation values for all relevant projector operators  and , taking care of the renormalization performed. (Figure 4d). (In practice, the electronic amplitudes are assimilated in the wave packet and are thus not extracted separately. See section S4 in the SI for further details.)

For what concerns the last point, we notice that, in principle, the expectation values need to be evaluated frequently enough (every few hundreds of attoseconds) to capture the relevant oscillatory features of the coupled electronic–vibrational dynamics. To circumvent this bottleneck, in practice, the energies of the *g* and *f* manifolds are uniformly shifted in order to minimize the electronic energy gap among all pairs of dipole-coupled states. This allows us to evaluate the overlap less frequently. The effect of this uniform (vertical) shift of the potential energy surfaces can be reintroduced as a constant frequency shift of the given contribution after the Fourier transform (eq 3). Note that this computational speed-up is practical only because by construction the *g*, *e*, and *f* manifolds are uncoupled.

The QD simulations are the time-consuming steps of the protocol. In practice, the spectra reported in the following sections are computed by performing QD simulations on the *e* manifold in the interval  for a grid of times *t*_2_ = *n*Δ*t* with Δ*t* = 2 fs and *n* = [0, 200]. Each of these runs was followed by a time evolution on the *e* + *f* manifold for time *t*_3_ = 100 fs. All of these computations take approximately 1 day on a Xeon processor with 52 cores. It is expected that in future work, adopting ML-MCTDH and similar computational resources, it will be possible to obtain spectra of systems with ∼10–15 electronic states and a few dozen normal modes within a few days.

In the current implementation, the electronic state lifetime broadening is naturally included through the time-dependent amplitudes. Dephasing (i.e., the decay of the signal due to the coupling to the environment) is, however, treated phenomenologically by multiplying the overlap term with a Gaussian that also assures that the nonlinear response function goes to zero with *t*_3_, facilitating a clean Fourier transform (eq 3). More details specific to the pyrene case are given in the Results section.

#### 2.5.2 Spectron

Computing the approximate expressions of eqs 17 and 19 does not require explicit dynamics simulations but solely the parametrization of the phase functions *g*_*ge*_(*t*) and φ_*cba*_(τ_4_, τ_3_, τ_2_, τ_1_). The ingredients needed are identical to the ones required to parametrize the LVC model Hamiltonian, except for the interstate couplings that are neglected. Instead, the population dynamics is accounted for by the rate matrix , which in turn requires preliminary knowledge (or a hypothesis) of the mechanism of IC (e.g., through experimentally available rates and lifetimes). In practice, the following workflow is adopted:

- iSpectron,^63^ an interface to several QM software packages, extracts normal modes, electronic energies, gradients, and TDMs in a semiautomatized way and builds state-specific and pair-state spectral densities;
- a development version of Spectron^7^ computes the line shape functions (through an integral transform of the spectral densities) which compose the phase functions in eqs 17 and 19;
- eq 19 is solved in equal intervals *t*_2_ (every few femtoseconds) to capture the relevant oscillatory features of the vibrational dynamics.

We note that typically the dephasing-induced signal broadening is not implemented via a Gaussian factor but via line-shape functions computed through a specified spectral density. Even if Spectron can handle spectral density of arbitrary shape, for simplicity we used the widely employed overdamped Brownian oscillator (OBO)^64,65^ model spectral density, which requires only a system bath coupling strength λ and cutoff frequency Λ to be defined.^63^ Despite being formally different, the signal broadening formalism used in the exact and approximate responses gives very similar line shapes.

## 3 Results

In this section, we present the results of simulations obtained for both LA and TA spectra at various levels of theory: (i) exact MCTDH, employing eqs 13 and 14, (ii) the MCTDH* model, employing eq 16 and eq 18, thereby separating dynamics of coherences from that of populations, and (iii) SPEC (analytic) model, employing eqs 17 and 19, which is the most approximate level considered here. We chose to study the ultrafast internal conversion in pyrene excited with near-UV light to its first bright state, labeled *S*_2_. The deactivation from *S*_2_ to the lower-lying long-lived dark *S*_1_ has been extensively studied both experimentally^66−69^ and theoretically,^33,70,71^ showing that the nonadiabatic decay occurs on a sub-100-fs time scale. Notably, such ultrafast decay is more a consequence of coupling between vibrational levels rather than ballistic motion toward a conical intersection, as the wave packet never reaches the crossing region.^33^

### 3.1 Parametrization of the LVC Hamiltonian

Multilayer MCTDH (ML-MCTDH) QD simulations with a full-dimensional LVC Hamiltonian (which included all symmetry-allowed modes out of the 72 normal modes, only 49 were considered, as the remaining 23 possess zero coupling and gradient for symmetry reasons) parametrized through QM calculations with OpenMolcas^72,73^ at the restricted active space self-consistent field level of theory with multireference second order perturbation correction of the energetics (i.e., RASSCF/RASPT2) have been recently reported by some of the authors.^33^ The model includes the lowest excited singlets, labeled *S*_1_ through *S*_7_, in the *e* manifold. Two of the states, *S*_2_ and *S*_5_, are spectroscopically bright and absorb in the near-UV (340–290 nm) and mid-UV (275–250 nm). In the present work, we utilize the single layer variant of MCTDH implemented in Quantics which offers a straightforward implementation of the algorithm described in the previous section. The adoption of single-layer MCTDH requires us to limit of the number of nuclear degrees of freedom. At equilibrium, pyrene has *D*_2*h*_ symmetry. As a consequence, one can differentiate between normal modes, termed either tuning or coupling, which give rise to intra- or interstate couplings, respectively. The intrastate couplings were computed numerically at the single-state (SS) RASPT2 level of theory on top of a restricted active space RAS(4, 8|0, 0|4, 16) in *D*_2*h*_ symmetry, whereas interstate couplings were computed with lower symmetry using the multistate (MS) variation of the RASPT2 method. The evaluation of the coupling strength along all modes was performed with a version of the Overdia code^74^ modified to read energies and overlaps computed with an external code (in this case, OpenMolcas), and it was shown that the obtained model accurately reproduces the QM potential energy surfaces in the coordinate region relevant for the QD.^33^ In the computations performed for the current work, we restricted the model to the 15 most relevant tuning and coupling modes to reduce the computational cost associated with running a substantial number of propagations. Specifically, 201 independent runs (of *t*_2_ + *t*_3_ duration) were initiated: the *t*_2_ waiting time ranges between 0 and 400 fs (every 2 fs), and the *t*_3_ time ranges from 0 to 100 fs (every 0.5 fs). Refer to section S2 of the SI for further details on the normal mode selection. A comparison with the full-dimensional dynamics (see Figure S1 in the SI), obtained with ML-MCTDH method, demonstrates that the reduction of the dimensionality affects the electronic dynamics only moderately.

The pump pulse was chosen to be resonant with *S*_2_. Accordingly, for the purpose of simulating TA spectroscopy on top of the *S*_2_ → *S*_1_ internal conversion, the model was supplemented with five higher-lying electronic states, comprising the *f* manifold, selected from a manifold of 40 electronic states of *A*_*g*_ and *B*_1*g*_ symmetry (20 states per symmetry) owing to their high TDMs to either *S*_2_ or *S*_1_. For the *f* manifold, we included only intrastate couplings along the tuning modes, computed from numerical gradients obtained at the same level of theory described above for the *e* manifold. It should be mentioned that our recent study^33^ suggests the involvement of higher-lying states *S*_3_–*S*_7_ as mediators for *S*_2_ → *S*_1_ population transfer. However, due to their strong coupling to both *S*_1_ and *S*_2_, they never acquire a significant transient population that could give rise to intense transient signals. For this reason, while we consider seven excited states in the nonadiabatic dynamics we limit the simulation of photoabsorption signals to the lowest two. Hence, the *f* manifold comprises only states dipole coupled to *S*_1_ or *S*_2_. Table 1 reports the energies and module of *g* – *e* and *e* – *f* TDM calculated at the reference geometry. We clarify that due to the nanosecond lifetime of the *S*_1_ state, the GS was not included in the dynamics. It enters in the expressions only through its light-induced coupling to *S*_2_ giving rise to SE.

**Table 1:** Irreducible Representations (*D*_2*h*_ Symmetry Group), Transition Dipole Moment (TDM) Magnitudes, and Vertical Excitation Energies for the Excited States Included in the Model Computed at the GS Equilibrium at the SS-RASPT2/RASSCF(4,8|0,0|4,16)/ANO-L-VDZP Level of Theory^a^

state	*D*_2_*_*h*_* irred. repr.	TDM (a.u.)	energy (eV)
**S**_0_	*A*_*g*_		0.00
*S*_1_	*B*_3*u*_	0.00 (*S*_0_)	3.23
*S*_2_	*B*_2*u*_	1.83 (*S*_0_)	3.75
*S*_3_	*B*_1*g*_	0.00 (*S*_0_)	4.16
*S*_4_	*A*_*g*_	0.00 (*S*_0_)	4.32
*S*_5_	*B*_3*u*_	1.73 (*S*_0_)	4.43
*S*_6_	*B*_1*g*_	0.00 (*S*_0_)	4.56
*S*_7_	*B*_1*g*_	0.00 (*S*_0_)	4.82
*S*_11_	*A*_*g*_	0.84 (*S*_1_)	5.62
*S*_14_	*B*_1*g*_	1.00 (*S*_1_)	5.84
*S*_15_	*A*_*g*_	2.10 (*S*_2_)	5.88
*S*_18_	*B*_1*g*_	1.25 (*S*_1_)	6.70
*S*_33_	*A*_*g*_	2.32 (*S*_2_)	7.56

a In parentheses, we report the state to which the TDM refers.

A model with a reduced number of electronic states (i.e., comprising only *S*_1_, *S*_2_, and *S*_3_) has also been considered. The seven states and the three state models are labeled as MCTDH(7st) and MCTDH(3st), respectively.

All MCTDH dynamics were run at 0 K, i.e., assuming that the system lies in the (global) ground vibrational state prior to photoexcitation. Since in pyrene the dynamics involves only (for symmetry reason) modes with frequency larger than 350 cm^–1^, whose Boltzmann population at room temperature is small, this is perfectly justified.

### 3.2 Population Dynamics with MCTDH

**Figure 5:**
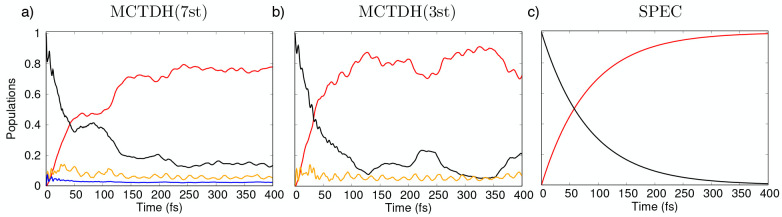
Pyrene population dynamics after *S*_2_ excitation: (a) the 7 states/15 modes MCTDH model, (b) the 3 states/15 modes MCTDH model, and (c) the purely exponential SPEC model from Pauli master equations. The initial population, set to 1 in the *S*_2_ state (black curve), rapidly converts to *S*_1_ (red curve) with a quasi-exponential decay. Note that both the *S*_2_ and *S*_1_ dynamics are affected by the *S*_3_ state (reported in orange). The sum of the transient population of all of the other states in the MCTDH(7st) model (reported in blue) is minor.

Figure 5a reports the pyrene population dynamics obtained with the 7 states/15 modes LVC model Hamiltonian described above. The dynamics is initiated with amplitude  (i.e., population entirely projected on the bright *S*_2_ at time zero). We observe a rapid conversion into *S*_1_ with an almost exponential decay modulated by a weak oscillation with a period of about 100 fs (red line). These oscillations are also present in the full-dimensional dynamics albeit being less pronounced (Figure S1 in the SI).^33^ After 150 fs, about two-thirds of the population has relaxed in *S*_1_. The remaining states of the *e* manifold show marginal populations. We note a coherent high-frequency oscillation (period of ca. 20 fs) of the *S*_3_ population. Similar dynamics is also observed for the 3 states/15 modes model, which presents a faster initial decay (see Figure 5b). Figure 5c presents the purely exponential dynamics resulting from solving the Pauli master equation for a rate matrix  with an off-diagonal value of  fs^–1^ (corresponding to a lifetime  fs chosen so as to match the experimental lifetime of *S*_2_(69)). The population dynamics allows to get an idea of the TA spectrum: signals from the bright *S*_2_ (ESA and SE) are expected to be intense in the first few tens of femtoseconds and to gradually diminish, leaving the floor to *S*_1_ ESA.

### 3.3 Response Functions: The Pyrene Case

The equations derived in section 2 can be simplified by taking into account the specific case of the pyrene molecule excited in the bright *S*_2_ state. For the first-order response, one obtains
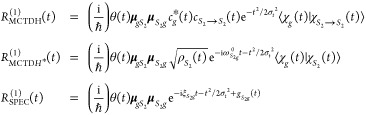
20The Feynman diagram representation is reported in Figure 2 (bottom). In particular, one notices that, by considering a spectral window in which there is only one bright state, the number of possible contributions that make up the first-order response is drastically reduced.

In the case of the third-order response, a great reduction of ESA terms is achieved since (a) by symmetry no state from the *f* manifold is dipole-coupled simultaneously to *S*_1_ and *S*_2_; (b) by construction of the model, interstate couplings and thus nonadiabatic dynamics in the *f* manifold are neglected. Furthermore, under the assumption that pump and probe pulses have the same polarization, one can use the scalar form (magnitude) of the TDMs instead of the vectorial form. Taking these considerations into account, one obtains the following equations for the nonlinear spectroscopy of pyrene:
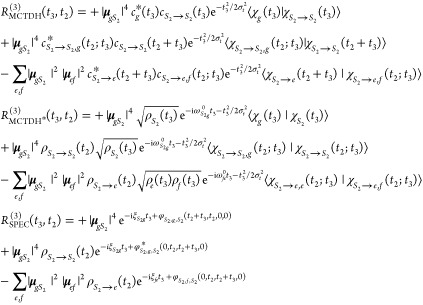
21The Feynman diagram representation is reported in Figure 3 (bottom). In the MCTDH expression, σ_*t*_ = 23.6 fs. In the SPEC expression, the phase functions φ contain both the DHO and the OBO contributions, with the latter using a uniform set of parameters λ = 150 cm^–1^ and Λ = 85 cm^–1^ for all states. In the SPEC expression, the lifetime broadening of the GSB and SE contributions was achieved by a time constant  fs (entering through ). For convenience, the same value was used for the lifetime broadening of all of the ESA contributions. A more proper description would require us to consider a different value for each contribution (entering through ξ_*fe*_), depending on the lifetimes of the pair of *e*, *f* states constituting the coherence.

### 3.4 Linear Absorption

**Figure 6:**
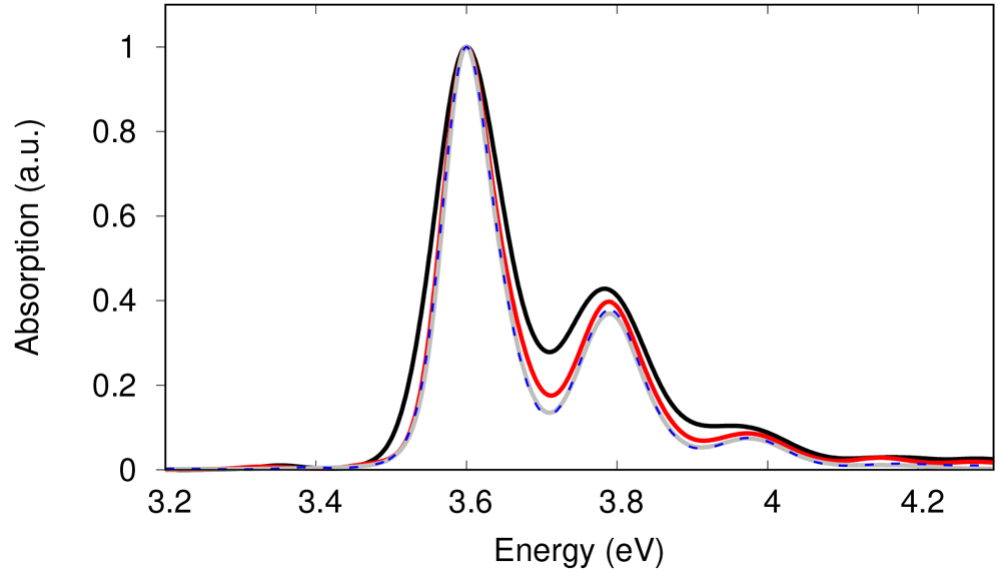
Comparison of the LA spectra obtained at different levels of theory: MCTDH(7st) (black), MCTDH(3st) (red), MCTDH*(7st) (dashed blue), and SPEC (gray). The spectra are normalized and aligned to the maximum of the fundamental band. Note that MCTDH*(7st) and SPEC spectra are extremely similar. MCTDH(7st) is notably broader than all of the others.

**Figure 7:**
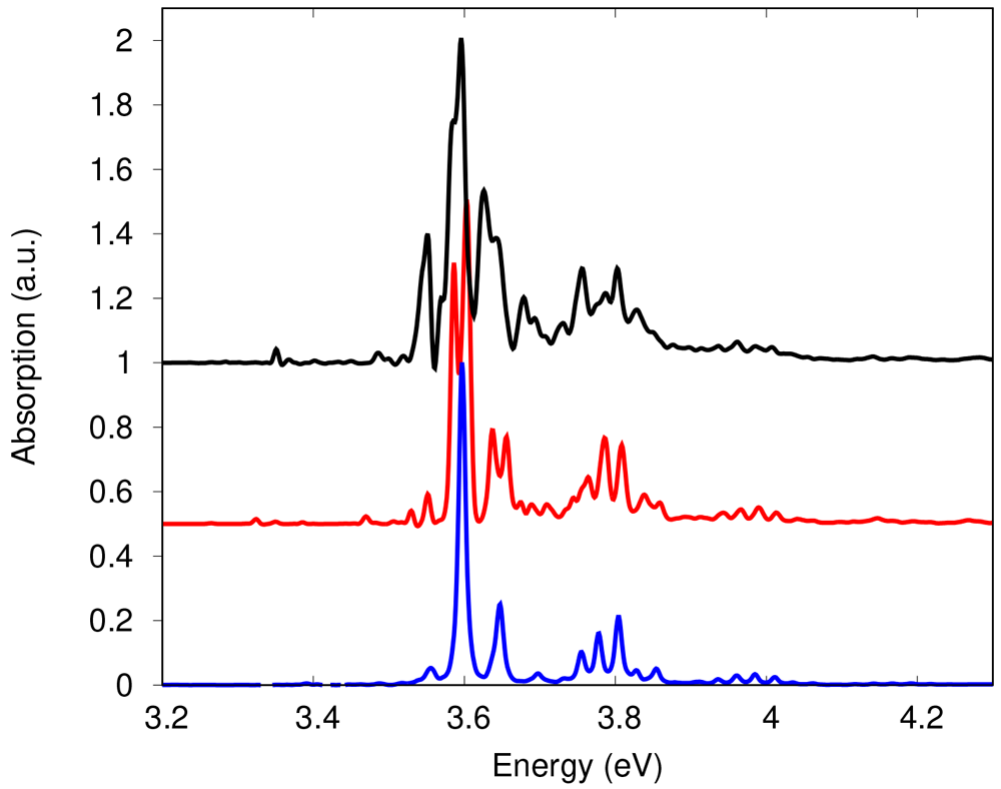
Comparison of the LA spectra obtained at different levels of theory and employing an extremely small dephasing factor (σ_*t*_ = 176.8 fs): MCTDH(7st) (black), MCTDH(3st) (red), and MCTDH*(7st) (blue) normalized to the height of the fundamental. The SPEC level is not shown as it almost exactly matches the MCTDH*(7st) spectrum. Note how the complexity and structure of the spectrum increase from MCTDH*(7st) to MCTDH(7st).

In Figure 6, we report the linear absorption spectra obtained with eqs 20. Note that the spectra are normalized and aligned to the maximum of the fundamental band. The unshifted spectra are reported in Figure S2 in the SI. The MCTDH(7st) (black) and SPEC (gray) spectra show similar vibrational progressions, yet the MCTDH(7st) spectrum is notably broader. To better understand the origin of the broadening, we consider the MCTDH*(7st) model, for which the nonadiabatic dynamics was switched off during *t*_3_. This confines the wave packet on the *S*_2_ state (as in the SPEC approximation), while one can still dress the response function a posteriori with the  term obtained from the MCTDH(7st) model dynamics (eq 18). The so-obtained spectrum (dashed blue line) is conceived to test the hypothesis that the line shape of the MCTDH(7st) spectrum is a consequence of the nonexponential decay in the exact dynamics, exhibiting a more rapid decay in particular during the first 100 fs (compare panels a and c of Figure 5). The close agreement of the MCTDH* and SPEC spectra demonstrates that the line shape is mainly dictated by the wave packet overlap term rather than by the lifetime broadening. This is further emphasized by strongly reducing the dephasing term in both MCTDH(7st) and SPEC expressions (corresponding to performing experiments at cryogenic temperature or in the gas phase). The MCTDH*(7st) spectrum (in blue in Figure 7) shows the expected multipeak structure due to the vibrational progressions in the totally symmetric modes included in the model: 405.5, 1271.4, 1456.1, and 1668.6 cm^–1^. The MCTDH(7st) spectrum (black in Figure 7) has a much richer structure, the source of which is the strong coupling between *S*_2_ and the higher-lying states. This is illustrated by simulating the LA spectrum with the MCTDH(3st) model described above. Remarkably, the resulting spectrum (red in Figure 6) is much narrower than the MCTDH(7st) one despite the *S*_2_ → *S*_1_ population transfer occurring at an even faster rate (Figure 5b). The efficient coupling of the mediator *S*_3_ to both *S*_2_ and *S*_1_ facilitates an ultrafast unidirectional population transfer (*S*_2_ lifetime  fs in this case), while the surfaces retain their harmonic nature, reflected in the LA line shape which exhibits only a fine splitting of some of the peaks (in red in Figure 7) with respect to the fully harmonic spectrum SPEC (in blue in Figure 7). The inclusion of further close-lying and strongly coupled states (*S*_3_–*S*_6_ lie all together in the energy range of 0.4–0.8 eV above *S*_2_)^33^ causes population to efficiently flow back to the *S*_2_ state, clearly visible in the 50–100 fs time interval, thus overall slowing down the population build up in *S*_1_ (MCTDH(7st) dynamics in Figure 6a). It appears that the wave packet returning to *S*_2_ is out of phase with the one still residing in *S*_2_. As a consequence, the overlap  exhibits a faster decay with irregular revivals, eventually causing the richer and broader features in the MCTDH(7st) spectrum (black line in Figure 7). A comparison between the overlaps for MCTDH(7st), MCTDH(3st), and MCTDH*(7st) is shown in the SI (section S7).

### 3.5 Transient Absorption

**Figure 8:**
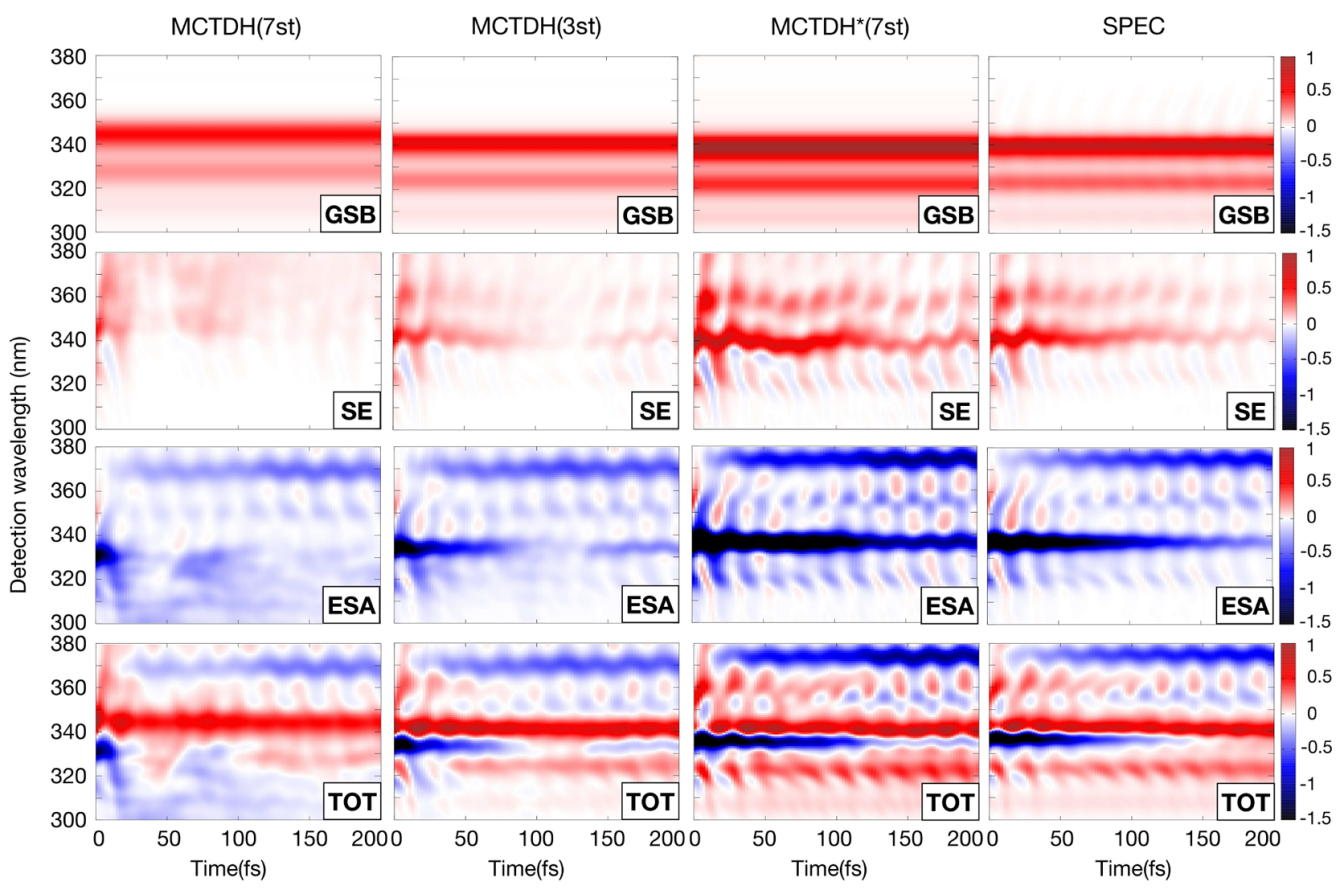
Comparison of the TA spectra obtained at different levels of theory: MCTDH(7st), MCTDH(3st), MCTDH*(7st), and SPEC. Note the more diffuse nature of the MCTDH(7st) signals due to the stronger coupling of *S*_2_ with the *S*_3_–*S*_7_ set of states; note also the almost identical results for MCTDH*(7st) and SPEC spectra.

The analysis for the LA line shapes suggests that also in the TA spectra the effect of the nonadiabatic dynamics will be nontrivial, with a significant impact on the spectral line shape. Figure 8 compares the MCTDH(7st), MCTDH(3st), MCTDH*(7st), and SPEC TA spectra. Thereby, individual maps for each of the different contributions—GSB, SE, and ESA—as well as the complete spectrum are reported. We note that in the TA setting the ESA has opposite sign (blue) to GSB and SE (red). The GSB comprises three bands correlated to the vibronic structure of the LA spectrum which show no time dependence. This behavior is expected as the GSB contribution has no *t*_2_ dependence, and its line shape is determined by the same overlap (MCTDH)/phase function (SPEC) as in the linear response. The SE, originating from *S*_2_, shows a vibronic structure along the detection axis (clearly identifiable in the MCTDH*(7st) and SPEC spectra) which mirrors that of the GSB and is accompanied by a *t*_2_-dependent decay modulated by intensity beating with an approximately 20 fs period. The ESA component shows two contributions, one that shows up instantaneously at 335 nm and decays on the same time scale as the SE and another one that rises with a delay at 370 nm. These two features belong to ESA signals from *S*_2_ and *S*_1_, respectively, and show quantum beating along *t*_2_.

We now analyze the differences among the various protocols. First, we note that the MCTDH(7st) maps are much more diffuse. Notably, it is the *S*_2_ features which are affected by the explicit consideration of nonadiabaticity during the coherence evolution. In particular, the SE and ESA from *S*_2_ are remarkably broad and less structured and disappear almost instantaneously. In fact, despite having still 40% of the population in *S*_2_ at around *t*_2_ = 50 fs, the SE and ESA signatures are nearly completely washed out. We note a weak signal revival between 50 and 100 fs, which correlates to the population revival (Figure 5a). We identify the strong vibronic coupling of *S*_2_ with the higher-lying states as the main culprit for the washing out of the transient *S*_2_ features. Removing states *S*_4_–*S*_7_ from the model (MCTDH(3st)) leads to more structured and longer-lived *S*_2_ signals (despite the shorter *S*_2_ lifetime in the MCTDH(3st) model), whereas turning off the interstate couplings during *t*_3_ (MCTDH*) confines the coherence dynamics to the harmonic regime, which translates into spectral signatures virtually indistinguishable from those of the SPEC spectrum.

**Figure 9:**
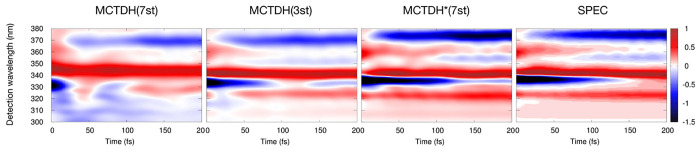
Same as for Figure 8, but taking into account the finite time resolution of a realistic experiment (i.e., convoluting the data with a Gaussian of σ = 8 fs (fwhm = 18.8 fs)).

Finally, it is worth mentioning that the computed spectra assume an infinite time resolution in the simulated experiment: in fact, the pronounced quantum beating along *t*_2_ with a 20 fs period would be partially washed out by the finite time resolution of a realistic experiment (due to the finite time duration of realistic pulses). This can be approximately recovered by convoluting the ideal spectra in time with a Gaussian whose standard deviation σ is related to the duration of the experimental pump pulse. Figure 9 shows the convoluted total spectra for the four protocols utilizing a Gaussian with σ = 8 fs (equivalent to fwhm = 18.8 fs). One can note that the high-frequency oscillations are no longer visible.

## 4 Conclusions

In this contribution, we presented a highly accurate and efficient quantum dynamics approach to simulating linear and nonlinear optical spectroscopy. We derived expressions for first- and third-order responses of a closed quantum system subject to an impulsive interaction with an incident electric field within the Condon approximation: these expressions (eqs 13 and 14) allow us to incorporate the effect of ultrafast nonadiabatic dynamics by performing explicit numerical propagation of the nuclear wave packet. In practice, the propagation is realized by means of the multiconfiguration time-dependent Hartree (MCTDH) approach through its single-layer formulation implemented in Quantics and capable of efficiently treating about one/two dozen nuclear degrees of freedom. The dynamics is driven by a linear vibronic coupling model Hamiltonian parametrized from high-quality QM data. The protocol paves the way for simulating UV/vis electronic spectroscopies such as linear or transient absorption (TA) for a number of ultrafast photoresponsive rigid systems such as polycyclic aromatic hydrocarbons, transition-metal complexes, and aromatic stacks.

Using the same formalism adopted for deriving the exact expressions, we recast a known approximation based on the time scale separation between dephasing (assumed fast) and population decay (assumed slow). This approximation allows us to omit explicit wave packet propagation and to derive analytical expressions for the molecular response functions (eqs 17 and 19) at the expense of memory erasure of previously visited states and a violation of energy conservation.

The (exact and approximate) protocols were applied to the pyrene molecule, a system that exhibits an ultrafast decay from its lowest bright *S*_2_ state to a dark *S*_1_ state completed within 100 fs. Pyrene was chosen in order to demonstrate the effect of ultrafast nonadiabatic processes on the spectral line shapes and to assess the fitness of the approximations. Remarkably, we find that the approximate solutions manage to reproduce the signal line shape fairly accurately even in the case of sub-100-fs nonadiabatic dynamics as long as interstate couplings do not introduce strong anharmonicities and population revival that could cause wave packet interference effects. These could lead to less-structured and faster-decaying signals. As a consequence, it can be envisioned that the approximate solution will not be suitable for simulating transient spectroscopy in systems with high densities of strongly coupled states such as metal–organic complexes, for example.

The proposed MCTDH-based implementation of nonlinear spectroscopy is completely general. Its application in practice depends on two parameters: the complexity of the electronic structure and the cost of numerical propagation. While we have demonstrated the feasibility of the protocol for small to medium-sized organic molecules, going toward larger systems (e.g., multichromophoric aggregates) would require us to consider a substantial increase in both the electronic and vibrational DOFs. Even if LVC is easily generalized to account for such a large number of states,^43,75^ this poses several new challenges: (a) Regarding the accuracy and computational cost of the electronic structure methods employed to parametrize the system Hamiltonian, while some of the present authors have shown that more than 100 excited states can be computed reliably at the RASSCF/RASPT2 level,^76−78^ novel techniques in the field of multiconfigurational wave function theory (such as the density matrix renormalization group (DMRG)^79^ or full configuration interaction quantum Monte Carlo (FCIQMC) approach^80^ combined with CASSCF) show promising results in solving the CI problem for a fraction of the computational cost). (b) Regarding the MCTDH dynamics itself, the single-layer variant of MCTDH arrives at its limits already with a dozen degrees of freedom. The problem of increasing dimensionality can be alleviated by switching to ML-MCTDH, which would allow for hundreds of degrees of freedom. Moreover, it has been shown that it suffices to consider weakly coupled modes at the Redfield level of theory so that only the most strongly coupled modes need to be explicitly included in the model.^81,82^ Note also that a more complex representation of the potential energy surfaces aimed at including anharmonicities (e.g., higher-order polynomials or grid-based representations) is possible with the MCTDH implementation in Quantics,^83^ at the expense of decreasing efficiency. (c) The presence of a dense manifold of states would require going beyond the closed-quantum system description, as bath-mediated transport would become relevant in this case. Here, we mimicked the presence of a system–bath coupling by introducing a phenomenological Gaussian dephasing term that acts in the postprocessing of the time-dependent overlaps (by damping their evolution). The sole effect of such a dephasing term is that of introducing a (Gaussian-shaped) homogeneous contribution to the total broadening, masking the detailed vibronic peak structure that would otherwise appear. This phenomenological term is meant to capture the detailed effects that are neglected when assuming a wave function formulation as opposed to a density matrix picture. Going beyond the closed-system approximation would require us to capture both static disorder (treating, for example, an ensemble of Hamiltonians that describe the many solvent configurations around each molecule in a sample) and dynamic disorder (e.g., coupling the explicit QD with some description of the bath, either via Redfield theory^81^ or evolving the solvent that surrounds the molecule according to Newton’s equation of motion coupled to the QD equations;^43^ another option might be to consider a large number of low-frequency bath modes within an ML-MCTDH QD treatment).

TA is not the only type of spectroscopic method that can be simulated with the current protocol. Extension of the technique in the X-ray regime is straightforward^84−86^ and will facilitate simulations of transient near-edge X-ray absorption fine structure (NEXAFS) spectra. The overlap term appearing in eqs 13 and 14 can be seen as the matrix element of the simplest operator (i.e., the identity operator). Evaluation of matrix elements of, for example, the polarizability and density operators will allow us to extend the proposed protocol for the simulation of more elaborate spectroscopy techniques, such as ultrafast X-ray Raman,^87^ time-resolved diffraction,^35^ and TRUECARS.^88^

Finally, accurate spectroscopy simulations would require the inclusion of realistic pulses. One option to account for finite-pulse duration is to postprocess the signals with the electric field envelop,^7^ as prescribed by eq 1. Another option goes in the direction of directly including the electric field in the MCTDH dynamics (i.e., adopting an explicit (and nonperturbative) approach), which has been already explored in the literature, as described in the Introduction.

## Supporting Information Available

Explicit expressions:  and ; LVC: additional details; MCTDH population dynamics: full vs reduced dimensionality model; MCTDH-spectroscopy implementation: practical notes; SPEC: line-shape functions; unshifted LA spectra; and overlaps comparison (PDF)
