# Supplementary material for: Nonlinear Molecular Electronic Spectroscopy via MCTDH Quantum Dynamics: From Exact to Approximate Expressions
Source: J Chem Theory Comput. 2023 Mar 24;19(7):2075–91. doi: 10.1021/acs.jctc.2c01059 (PMC10100531; doi:10.1021/acs.jctc.2c01059)
Supplement: Supplementary file 1 — ct2c01059_si_001.pdf [file ct2c01059_si_001.pdf]

SUPPORTING INFORMATION FOR

Nonlinear molecular electronic spectroscopy via MCTDH quantum dynamics: from exact to approximate expressions

Francesco Segatta,[*,†,‡] Daniel Aranda Ruiz,[†,¶,§] Flavia Aleotti,[†,‖] Martha Yaghoubi,[†,§] Shaul Mukamel,[⊥] Marco Garavelli,[*,‡] Fabrizio Santoro,[*,§] and Artur Nenov[*,‡]

†*contributed equally*

‡*Dipartimento di Chimica Industriale "Toso Montanari", University of Bologna, Viale del Risorgimento, 4, 40136 Bologna, Italy*

¶ *ICMol, Universidad de Valencia, Catedrático José Beltrán Martínez, 2, 46980 Paterna, Spain*

§*Istituto di Chimica dei Composti Organometallici (ICCOM-CNR), Area della Ricerca del CNR, Via Moruzzi 1, I-56124 Pisa, Italy*

‖*Dipartimento di Chimica Industriale "Toso Montanari", University of Bologna, Viale del Risorgimento, 4, 40136 Bologna, Italy*

⊥*Department of Chemistry and Department of Physics and Astronomy, University of California, Irvine, 92697, USA*

E-mail: francesco.segatta@unibo.it; marco.garavelli@unibo.it; fabrizio.santoro@pi.iccom.cnr.it; artur.nenov@unibo.it

This PDF includes:

- Explicit expressions: $\hat{T}_{ab}$ and $\hat{V}_{ab}(Q)$;
- LVC: additional details;
- MCTDH population dynamics: full vs reduced dimensionality model;
- MCTDH-spectroscopy implementation: practical notes;
- SPEC: line-shape functions;
- Unshifted LA spectra;
- Overlaps comparison;

S1 Explicit expressions: $\hat{T}_{ab}$ and $\hat{V}_{ab}(Q)$

The explicit expressions for the kinetic ($\hat{T}_{ab}$) and the potential ($\hat{V}_{ab}(Q)$) operators in the *diabatic* basis are here reported.

$$T_{ab} = \frac{1}{2}\mathbf{P}^T\mathbf{\Omega}\mathbf{P}\cdot\delta_{ab} \tag{S1}$$

$$V_{aa}(\mathbf{Q}) = E_a^0 + \boldsymbol{\lambda}_{aa}^T\mathbf{Q} + \frac{1}{2}\mathbf{q}^T\mathbf{\Omega}\mathbf{Q}, \tag{S2}$$

$$V_{ab}(\mathbf{Q}) = \boldsymbol{\lambda}_{ab}^T\mathbf{Q} \tag{S3}$$

$\mathbf{P}$ is the associated momenta of normal coordinates $\mathbf{Q}$, δ_{ab} is Kronecker delta function, E_a^0 is the vertical excitation energy of ath excited-state at the g equilibrium geometry, $\boldsymbol{\lambda_{aa}}$ represents the intra-state coupling (gradients) of state a, $\boldsymbol{\lambda_{ab}}$ the inter-state coupling between states a and $b \neq a$ and finally, $\mathbf{\Omega}$ is the diagonal matrix of the vibrational frequencies which in the LVC model are defined identical to those of state g.

As described in the main text, $\boldsymbol{\lambda_{ab}}$ was considered exclusively for the e set of states and zero otherwise. On the contrary, $\boldsymbol{\lambda_{aa}}$ were computed for both e and f sets.

S2 LVC: additional details

The Linear Vibronic Coupling (LVC) Hamiltonian for quantum dynamics was setup for pyrene as documented by some of the authors in reference 1, but with two main differences. On one hand, the number of electronic states was enlarged to include in the model the f manifold (which consists of states S_{11}, S_{14}, S_{15}, S_{18} and S_{33} at the SS-RASPT2/RASSCF(4,8|0,0|4,16)/ANO-L-VDZP level of theory) that were added to the S_1-S_7 states. On the other hand, the implementation of wavepacket overlap calculations along the dynamics was only possible for MCTDH (and not for its multilayer version ML-MCTDH), and this imposed to reduce the number of nuclear coordinates: after analysis of our previous dynamics[1] we selected a group of 15 MP2/ANO-L-VDZP pyrene normal modes (Table S1) that showed diabatic S1-S7 cou-

plings and/or gradients in the final Hamiltonian higher than 0.08 eV in absolute value. It is noteworthy that this might lead to the exclusion of some modes bearing a significant component of the gradient for the second excitation manifold states, however, we decided to give a higher priority to the states responsible for the non-adiabatic dynamics (i.e., S_1-S_7).

Table S1: Normal modes of pyrene included in the LVC model.

**Mode**	**D_{2h} irred. repr.**	**Frequency (cm^{-1})**	**Red. mass (AMU)**
8	A_g	405.5	8.0252
26	A_g	798.9	5.7383
37	A_g	1079.3	2.0432
47	A_g	1271.4	2.6830
52	A_g	1456.1	8.1436
62	A_g	1668.6	7.9339
20	B_{2u}	690.9	6.9743
27	B_{2u}	814.6	4.5545
53	B_{2u}	1488.9	6.0788
6	B_{3u}	350.4	4.5443
15	B_{3u}	539.1	7.0622
54	B_{3u}	1506.7	8.5694
55	B_{1g}	1514.0	6.0195
56	B_{1g}	1519.5	5.3331
60	B_{1g}	1627.7	8.2909

The MP2/ANO-L-VDZP S_0 minimum was identified as reference geometry for the parameterization and the corresponding adiabatic states used as reference states for the subsequent maximum-overlap diabatization procedure (i.e., adiabatic states at displaced geometries were rotated so as to maximize their overlap with the reference states). The S_0-S_7 states were parameterized along the selected modes using single-state (SS) and multi-state (MS) RASPT2/RASSCF/ANO-L-VDZP calculations exactly as described in reference 1 (see corresponding main text and supplementary material for computational details, symmetry arguments and a graphical representation of the active space orbitals). In contrast, states in the second excitation manifold S_{11}, S_{14}, S_{15}, S_{18} and S_{33} were assumed to be uncoupled (i.e., all the corresponding off-diagonal terms in the Hamiltonian were set to zero and the diabatic and adiabatic representations coincide at all geometries), therefore they were only parameterized according to their vertical excitation energy and gradient at the

reference geometry. Vertical excitations and gradients of all states were calculated at the SS-RASPT2/RASSCF(4,8|0,0|4,16)/ANO-L-VDZP/D_{2h} level of theory.The possible overestimation/underestimation of state energies even for small displacements (as a consequence of mixing/crossing of the reference RASSCF states) could be mitigated by application of MS-RASPT2 variations, however, this would be computationally very demanding, and RASSCF gradients were used as a first approximation.

Table S2: Selected normal mode and their frequency for reduced dimensionality LVC model for the MS parametrization.

	MS	
Modes	Frequency	Modes number in 49 model
01	350.361	1
02	405.550	2
03	539.117	6
04	698.871	8
05	798.881	10
06	814.609	11
07	1079.300	14
08	1271.414	24
09	1456.125	29
10	1488.876	30
11	1506.719	31
12	1513.975	32
13	1519.488	33
14	1627.737	37
15	1668.564	39

S3 MCTDH population dynamics: full vs reduced dimensionality model

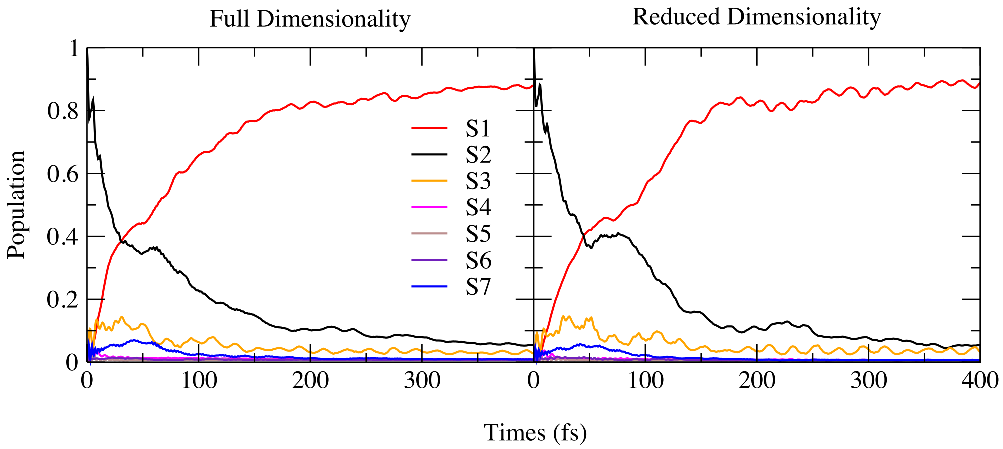

Figure s1: Dynamics of the populations of the diabatic electronic states obtained by initially exciting the wavepacket on S_2 (1B2u) for the MCTDH(7st) parameterization for full dymentionality on the left and reduced dimensionality on the right panels.

S4 MCTDH-spectroscopy implementation: practical notes

Consider Eq. 20 in the main text. It reads (in a bit more compact notation):

$$R^{(1)}_{MCTDH}(t) = \left(\frac{i}{\hbar}\right)\theta(t)|\boldsymbol{\mu}_{gS_2}|^2 c^*_g(t)c_{S_2\to S_2}(t)e^{-t^2/2\sigma_t^2}\langle\chi_g(0)\,|\chi_{S_2\to S_2}(t)\rangle \tag{S4}$$

First, as already noted in the main text, the wave-packet overlap terms obtained in MCTDH equals the product of the electronic amplitudes $c^*_g(t)c_{S_2\to S_2}(t)$ with the nuclear wave-function overlap $\langle\chi_g(0)\,|\chi_{S_2\to S_2}(t)\rangle$. Therefore we can rename the actual quantity extracted from the MCTDH dynamics as $\Xi_{gS_2}(t) = \theta(t)c^*_g(t)c_{S_2\to S_2}(t)\langle\chi_g(0)\,|\chi_{S_2\to S_2}(t)\rangle$ (where we have included also the Heaviside theta function, as $\Xi_{gS_2}(t)$ is computed from $t = 0$ on). Eq. S4 can be rewritten as:

$$R^{(1)}_{MCTDH}(t) = \left(\frac{i}{\hbar}\right)|\boldsymbol{\mu}_{gS_2}|^2 e^{-t^2/2\sigma_t^2}\Xi_{gS_2}(t) \tag{S5}$$

At this point, $R^{(1)}_{MCTDH}(t)$ is processed via the FT step. We recall that, in order to reduce the sampling frequency, a uniform (vertical) shift of the potential energy surfaces can be applied to reduce the $g - S_2$ energy gap by a factor $\bar{\omega}_{S_2g}$. The *correct* energy gap can be reintroduced by shifting the FT of the same $\bar{\omega}_{S_2g}$ factor. This corresponds to perform (without any approximation):

$$\begin{aligned} S^{(1)}(\Omega) &= \Im \int R^{(1)}_{MCTDH}(t) e^{i(\Omega - \bar{\omega}_{S_2g})t} dt \\ &\propto |\boldsymbol{\mu}_{S_2g}|^2 \Re \int \Xi_{S_2g}(t) e^{i(\Omega - \bar{\omega}_{S_2g})t - t^2/2\sigma_t^2} dt \end{aligned} \tag{S6}$$

Similar steps can be repeated for the third-order response, leading to the final equation.

$$\begin{aligned} S^{(3)}(\Omega, t_2) &= +|\boldsymbol{\mu}_{S_2g}|^4 \Re \int \Xi^{GSB}_{S_2g}(t_3) e^{i(\Omega - \bar{\omega}_{S_2g})t_3 - t_3^2/2\sigma_t^2} dt_3 \\ &\quad + |\boldsymbol{\mu}_{S_2g}|^4 \Re \int \Xi^{SE}_{S_2 \to S_2;g}(t_2, t_3) e^{i(\Omega - \bar{\omega}_{S_2g})t_3 - t_3^2/2\sigma_t^2} dt_3 \\ &\quad - \sum_{e,f} |\boldsymbol{\mu}_{gS_2}|^2 |\boldsymbol{\mu}_{ef}|^2 \Re \int \Xi^{ESA}_{S_2 \to e;f}(t_2, t_3) e^{i(\Omega - \bar{\omega}_{fe})t_3 - t_3^2/2\sigma_t^2} dt_3 \end{aligned} \tag{S7}$$

In practice, the $\bar{\omega}_{ab}$ shifts correspond to the vertical transition energy. The t time of the first-order response and the t_3 time of the third-order one were sampled every 0.5 fs, while the t_2 time was sampled every 2 fs. The integrals were performed numerically by means of the trapezoidal rule.

S5 SPEC: line-shape functions

$\varphi_{cba}(\tau_4, \tau_3, \tau_2, \tau_1)$ are the multidimensional phase-functions encoding the overlaps of WP moving on different adiabatic surfaces at different times. These are given by:

$$\begin{aligned}\varphi_{cba}(\tau_4,\tau_3,\tau_2,\tau_1) = \quad & -g_{cc}(\tau_{43}) - g_{bb}(\tau_{32}) - g_{aa}(\tau_{21}) \\ & -g_{cb}(\tau_{42}) + g_{cb}(\tau_{43}) + g_{cb}(\tau_{32}) \\ & -g_{ca}(\tau_{41}) + g_{ca}(\tau_{42}) + g_{ca}(\tau_{31}) - g_{ca}(\tau_{32}) \\ & -g_{ba}(\tau_{31}) + g_{ba}(\tau_{32}) + g_{ba}(\tau_{21}) \end{aligned} \tag{S8}$$

where $\tau_{ij} = \tau_i - \tau_j$. In the above expression $g_{ab}(t)$ are the so called line-shape functions which are integral transformation of the autocorrelation function describing the undamped oscillatory dynamics on the k-th normal mode harmonic potential with frequency ω_k and relative displacement with respect to the ground state equilibrium $\tilde{d}_{ak}$ and $\tilde{d}_{bk}$ in the a-th and b-th electronic state, respectively.

$$g_{ab}(t) = \frac{1}{2\pi} \sum_k \frac{\omega_k \tilde{d}_{a,k} \tilde{d}_{b,k}}{2} \left[\coth \frac{\hbar\omega_k}{2k_B T}(1 - \cos\omega_k t) + i \sin\omega_k t - i\omega_k t \right] \tag{S9}$$

As $\tilde{d}_{g,k} = 0$ by definition (i.e. GS equilibrium is at the origin) only the term $-g_{e'e'}(t_3)$ survives in the phase function for the GSB thus demonstrating that this pathway is t_2-independent and has a line shape resembling that of the linear absorption signal. The expressions for the SE and ESA signals are more complicated and carry the memory of the correlations between vibrational WP residing on different electronic states along each given mode during the intervals t_2 and t_3.

The state-specific line shape functions $g_{ab}(t)$ are parametrized employing similar inputs to the ones needed for the LVC model Hamiltonian: the normal mode frequencies ω_k and displacements $\tilde{d}_{ik}$. In practice, all these quantities can be obtained through QM calculations at one or more reference geometries.[2]

In the SPEC expressions the dephasing induced signal broadening is implemented via the line shape function of the overdamped Brownian oscillator (OBO) in the high temperature limit[3,4]

$$g_{ab}^{OBO}(t) = \frac{\lambda_{ab}}{\Lambda}\left(\frac{2k_BT}{\hbar\Lambda}(e^{-\Lambda t} + \Lambda t - 1)\right) \tag{S10}$$

with λ_{ab} and Λ the system-environment coupling strength and the fluctuation time scale. Thus, the total line shape function contains two components, one describing the vibrational structure of the signal (eq. S9) and a second one responsible for the solvent-induced homogeneous broadening (eq. S10).

S6 Unshifted LA spectra

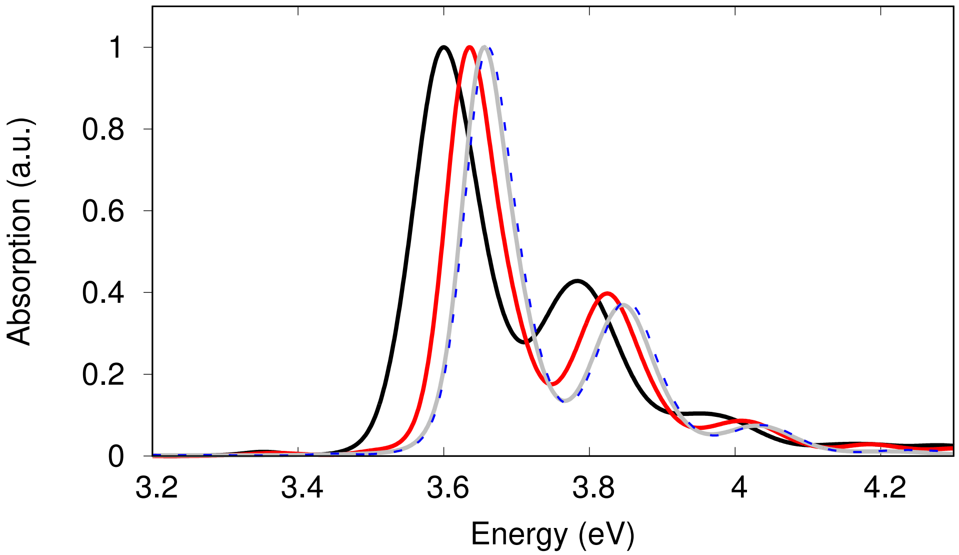

Figure s2: Comparison of the (unshifted) LA spectra obtained at different levels of theory: MCTDH(7st) (black), MCTDH(3st) (red), MCTDH*(7st) (dashed blue) and SPEC (gray). The spectra are normalized. The shifts between the fundamental bands of the different spectra with respect to the MCTDH(7st) level of theory are: 0.04 eV, 0.06 eV and 0.06 eV, for MCTDH(3st), MCTDH*(7st) and SPEC, respectively.

S7 Overlaps comparison

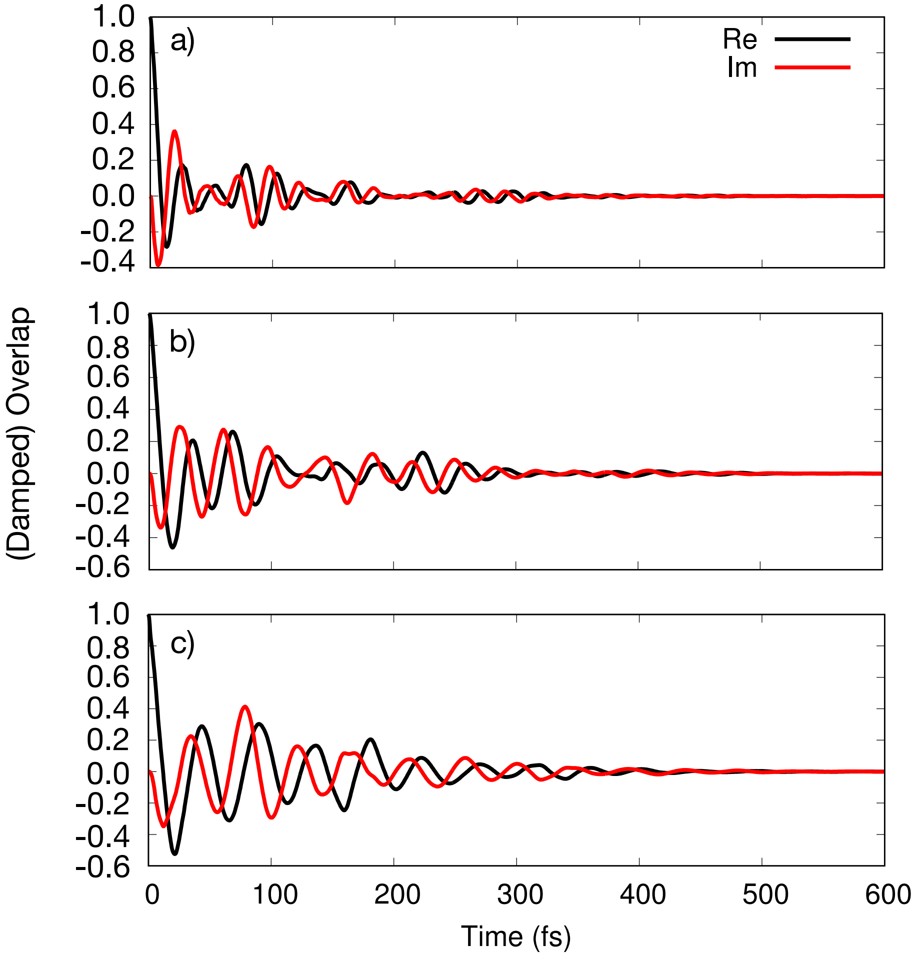

Figure s3: Comparison of the overlaps (damped with the the Gaussian dephasing factor, here set in the weak dephasing limit, $\sigma_t = 176.8$ fs) at different levels of theory: a) MCTDH(7st), b) MCTDH(3st) and c) MCTDH*(7st). Both real (black) and imaginary (red) parts of the overlaps are reported. Note that here the reported overlap also absorbs the $c_a(t)$ electronic coefficients.

References

(1) Aleotti, F.; Aranda, D.; Jouybari, M. Y.; Garavelli, M.; Nenov, A.; Santoro, F. Parameterization of a linear vibronic coupling model with multiconfigurational electronic structure methods to study the quantum dynamics of photoexcited pyrene. *The Journal of Chemical Physics* **2021**, *154*, 104106.

(2) Segatta, F.; Nenov, A.; Nascimento, D. R.; Govind, N.; Mukamel, S.; Garavelli, M. iSPECTRON: A simulation interface for linear and nonlinear spectra with ab-initio quantum chemistry software. *Journal of Computational Chemistry* **2021**, *42*, 644–659.

(3) Bosma, W. B.; Yan, Y. J.; Mukamel, S. Impulsive pump-probe and photon-echo spectroscopies of dye molecules in condensed phases. *Phys. Rev. A* **1990**, *42*, 6920–6923.

(4) Li, B.; Johnson, A. E.; Mukamel, S.; Myers, A. B. The Brownian oscillator model for solvation effects in spontaneous light emission and their relationship to electron transfer. *Journal of the American Chemical Society* **1994**, *116*, 11039–11047.
